# Fluid Resuscitation with Lactated Ringer vs. Normal Saline in Acute Pancreatitis: A Systematic Review and Meta-Analysis of Clinical Trials

**DOI:** 10.3390/diseases13090300

**Published:** 2025-09-10

**Authors:** Freiser Eceomo Cruz Mosquera, Elizabeth Camacho Benítez, Mariatta Catalina Ceballos Benavides, Julián Esteban Castillo Muñoz, Carlos Andrés Castañeda, Yamil Liscano

**Affiliations:** Grupo de Investigación en Salud Integral (GISI), Department of Health Sciences Faculty, Universidad Santiago de Cali, Cali 760035, Colombia; eli.cb0327@gmail.com (E.C.B.); catalinabenavidesr@gmail.com (M.C.C.B.); .; carlosandresc12345@gmail.com (C.A.C.); yamil.liscano00@usc.edu.co (Y.L.)

**Keywords:** acute pancreatitis, lactated ringer’s solution, normal saline, mortality, critical care

## Abstract

Background: Initial fluid therapy in acute pancreatitis is critical for modulating the systemic inflammatory response. The choice between Lactated Ringer and normal saline remains debated, given their potentially divergent impacts on disease progression and clinically relevant outcomes. The objective of this meta-analysis is to determine the effectiveness of one solution versus the other in patients with AP. Methods: A systematic review of randomized clinical trials published between 2000 and 2024 was conducted through an exhaustive search in databases such as PubMed, ScienceDirect, LILACS, SCOPUS, Web of Science, Springer, Scielo, and Cochrane. The review protocol adhered to the recommendations established by PRISMA. The methodological quality of the selected studies was assessed using the Jadad scale, while statistical analyses were performed with RevMan 5.4^®^ and Jamovi 2.3.28^®^ software. Results: Five trials with 299 patients showed that, in patients with AP, Lactated Ringer significantly reduced ICU admission (RR: 0.39; 95% CI: 0.18–0.85; *p* = 0.02) and the progression of pancreatitis (RR: 0.63; 95% CI: 0.40–0.98; *p* = 0.04). There was no significant difference in mortality or hospital stay (SMD: −0.89; 95% CI: −2.26 to 0.48; *p* = 0.23). No clear effects were observed on SIRS at 24, 48, and 72 h. CRP at 48 h was significantly lower with lactate (SMD: −3.91; 95% CI: −4.66 to −3.17; *p* < 0.00001), but not at 72 h. Conclusions: The administration of Lactated Ringer in acute pancreatitis shows clinical and anti-inflammatory benefits, but the evidence is mostly of low quality.

## 1. Introduction

Acute pancreatitis is one of the leading causes of gastrointestinal hospitalization and is characterized by the inappropriate intrapancreatic activation of digestive enzymes, with autodigestion, local inflammation, and a possible systemic inflammatory response with multiorgan dysfunction [1,2]. Although most cases are mild, up to 20% progress to moderate or severe forms with pancreatic necrosis, infection, and sepsis [3].

The initial management of AP is based on life support, clinical monitoring, and the prevention of early complications. One of the therapeutic pillars is moderate resuscitation with intravenous fluids, as its administration has been associated with a lower incidence of complications and higher survival [4,5]. Controlled fluid administration has proven to be essential in the treatment of AP, by reducing the risk of fluid overload while preventing or reversing hypovolemia induced by fluid extravasation into the retroperitoneal space [6]. However, despite the consensus on the importance of this therapeutic practice, controversy persists regarding which crystalloid solution offers better clinical outcomes. Still, crystalloids are preferred over colloids due to their greater safety, lower cost, and lower risk of adverse effects, such as acute kidney injury [7,8].

Traditionally, normal saline (NS) has been the most used in clinical practice. However, in recent years, Lactated Ringer (LR) has gained relevance due to its lower risk of inducing hyperchloremic acidosis and having a pH closer to that of plasma [9]. These properties confer protective effects on renal function and pancreatic tissue, as experimental studies have shown that pancreatic zymogens are activated in low pH conditions [10]. This suggests that the use of LR, compared to NS, could be associated with a better prognosis in patients with AP [11]. Experimental studies have suggested that LR could exert an immunomodulatory effect by down-regulating Toll-like receptor-induced NLRP3 inflammasome activation, which reduces the production of interleukin-1β (IL-1β) [12,13]. On the other hand, the administration of LR, compared to NS, has been associated with a lower incidence of systemic inflammatory response syndrome (SIRS). Likewise, some authors have documented that the use of LR has been linked to a significant decrease in C-reactive protein levels, a biomarker related to the severity of acute pancreatitis [14]. This reduction could help prevent the progression of the disease to severe forms and improve clinical outcomes [15,16,17,18,19].

Regarding the effects according to the type of crystalloid used, Aboelsoud et al. [20] reported a reduction in mortality in patients treated with LR compared to NS. However, other studies report that there is no statistically significant decrease in mortality, with the 30-day mortality being 10.3% in the crystalloid group and 11.1% in the saline group (*p* = 0.06) [21]. This reflects the current divergences in the evidence and the lack of consensus on the overall effect of one solution versus the other.

Although previous meta-analyses have compared the use of Lactated Ringer solution versus normal saline for the treatment of acute pancreatitis, some of these combine studies with different methodological designs and age groups. These characteristics make a homogeneous interpretation that is applicable to specific populations difficult. For this reason, the present systematic review and meta-analysis aims to evaluate the efficacy of using Lactated Ringer in comparison with normal saline in adult patients with acute pancreatitis.

## 2. Materials and Methods

### 2.1. Study Protocol

This systematic review and meta-analysis was developed in accordance with the Preferred Reporting Items for Systematic Reviews and Meta-Analyses (PRISMA) statement and the Cochrane Collaboration Handbook [22,23]. To ensure transparency, the research protocol was registered in the international PROSPERO database under the identification code CRD420251011996. The study’s design was structured based on the PICO (Population, Intervention, Comparison, and Outcomes) framework.

### 2.2. Research Question

In patients with acute pancreatitis, what is the effectiveness of fluid resuscitation with Lactated Ringer compared to normal saline in terms of hospital stay, ICU admission, development of systemic inflammatory response syndrome, progression to moderately severe or severe pancreatitis, C-reactive protein values, mortality, and adverse effects?

### 2.3. Eligibility Criteria

#### 2.3.1. Inclusion Criteria

Studies were considered for the review if they met the following requirements:

- Randomized controlled trials, regardless of the specific design (e.g., parallel, crossover) or follow-up duration.
- Publications spanning from the creation date of each database until February 2025, with no language restrictions.
- Studies conducted in adult patients diagnosed with acute pancreatitis that compared the effectiveness of fluid resuscitation with Lactated Ringer versus normal saline, regardless of the volume administered or the duration of therapy.
- Trials with multiple treatment arms where the Lactated Ringer intervention group could be isolated.
- Research reporting at least one of the following outcomes: days of hospitalization, ICU admission, presence of SIRS (at 24, 48, or 72 h), progression of pancreatitis severity, CRP levels, mortality, or adverse events.

#### 2.3.2. Exclusion Criteria

Investigations were excluded if they were

- Conference abstracts, articles in preprint format, or letters to the editor.
- Studies for which the full text was not available in an accessible format.
- Publications that used the same patient cohort as previously included clinical trials for the same purpose.
- Research that did not provide sufficient data for the calculation of the effect estimate.
- Studies conducted in animal models.

### 2.4. Data Sources and Search Strategy

A comprehensive search for scientific evidence was conducted in the Pubmed, LILACS, Science Direct, Springer, Scielo, SCOPUS, Web of Science, and Cochrane Clinical Trials databases. The strategy was designed and executed independently by three researchers (F.E.C.M., E.C.B., M.C.C.B.) between March and April 2025.

The search combined terms related to the intervention (“Lactated Ringer’s,” “Ringer lactate”), the comparison (“Normal saline,” “Saline Solution”), the condition (“Acute pancreatitis”), and the study type (“Randomized Clinical Trial”), while excluding reviews and observational studies. This strategy was adapted to the specifications of each database. As supplementary methods, the reference lists of relevant articles were reviewed, and a manual search was performed to identify additional studies. For supplementary information, the ClinicalTrials.gov registry was consulted. The Rayyan–Intelligent Systematic Review software was used for managing the records.

### 2.5. Study Selection and Data Extraction

The selection of articles was carried out by two researchers (J.E.C.M., C.A.C.) independently, who first evaluated the title and abstract, and subsequently the full text. If an article was inaccessible, an attempt was made to contact the corresponding author. Discrepancies regarding the inclusion of a study were resolved by consensus; if disagreement persisted, a third reviewer (Y.L.) was consulted to make the final decision. The Cohen’s Kappa index was used to measure the degree of inter-rater agreement.

Data extraction from the selected studies was performed by two reviewers (M.C.C.B. and E.C.B.), who collected information on: study characteristics (primary author, year, country), participant characteristics (number, age, sex, etiology, and severity of pancreatitis), intervention details (solution type, volume, timing, and duration), and the outcomes evaluated. Subsequently, a team of three reviewers (Y.L., F.E.C.M., and C.A.C.) verified the integrity and accuracy of the extracted data.

### 2.6. Risk of Bias Assessment

The risk of bias in the clinical trials was assessed independently by reviewers F.E.C.M. and J.E.C.M. using a standardized tool that covers key domains for this type of design [24]. The evaluated criteria included: random sequence generation, allocation concealment, blinding of participants and personnel, blinding of outcome assessment, management of incomplete outcome data, and selective reporting. For each domain, studies were classified as having a low, unclear, or high risk of bias. Differences in opinion between the reviewers were resolved through discussion until a consensus was reached. The information was recorded in Review Manager (RevMan) software, version 5.4^®^.

### 2.7. Assessment of Evidence Quality

The Jadad scale was used to evaluate the methodological quality of the studies, assigning a score from 0 to 5. This scale assesses five aspects: (a) whether the study was described as randomized, (b) whether a double-blind design was applied, (c) whether dropouts and withdrawals were reported, (d) whether the randomization method was appropriate, and (e) whether the inclusion/exclusion criteria were clear. While the quality score was not used as a criterion for excluding studies, it was considered during the analysis and interpretation of the findings.

### 2.8. Statistical Analysis

The meta-analysis was performed using RevMan 5.4^®^ software. A quantitative analysis was conducted when at least two studies evaluated the same outcome. The effect size was calculated with its 95% confidence interval (CI).

For dichotomous outcomes, the risk ratio (RR) was used, while for quantitative outcomes, the mean difference (MD) or the standardized mean difference (SMD) was used if measurement scales varied. A continuity correction was applied in trials with zero events in one or both groups. For the outcomes of mortality and adverse events, a qualitative synthesis was performed due to variability in their reporting.

Statistical heterogeneity was assessed with the I^2^ statistic. It was considered high if its value was 50% or greater. A fixed-effect model was applied if I^2^ < 50%, and a random-effects model was used if I^2^ ≥ 50%. A statistical significance threshold was set at *p* < 0.05. Subgroup analyses were planned based on resuscitation volume, initial severity, therapy start time, and pancreatitis etiology.

Publication bias was explored visually using funnel plots and statistically with Egger’s test, using Jamovi^®^ software. Finally, the certainty of the evidence was assessed with the GRADE (Grading of Recommendations, Assessment, Development, and Evaluation) system, considering domains such as risk of bias, inconsistency, and imprecision. The results were presented in a Summary of Findings (SoF) table.

## 3. Results

### 3.1. Studies Identified for the Review

In total, 885 records were identified through various search strategies: 673 records were retrieved from electronic databases, while 212 additional records were obtained through website searches and review of citations or bibliographic references.

After removing 143 duplicate records, 530 titles and abstracts were examined, with 470 being excluded for not meeting the criteria (Cohen’s Kappa: 90%). The full text of 60 articles was reviewed, but 16 could not be retrieved. Of the 44 full-text articles evaluated, 40 were excluded for the following reasons: they were not randomized clinical trials (*n* = 21), they were trial protocols (*n* = 10), they used animal models (*n* = 1), or the type of population was not relevant to the review’s objectives (*n* = 8) (Cohen’s Kappa: 92%).

**Figure 1:**
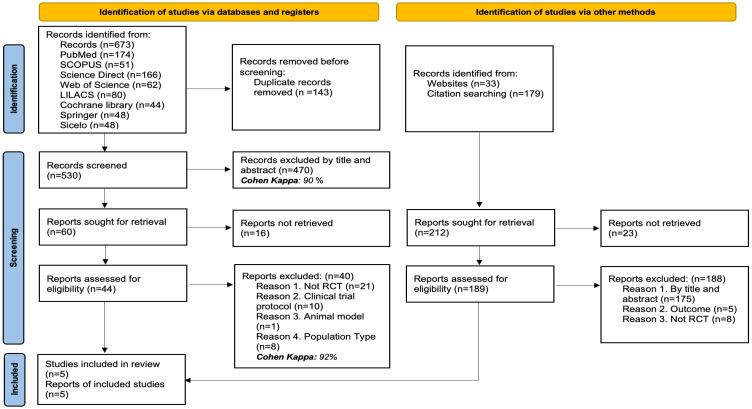
PRISMA flow diagram with the search and study selection strategy.

Simultaneously, 212 additional records identified through other methods were reviewed. Of these, 23 could not be retrieved. The remaining 189 were evaluated in full text, and 188 were excluded for the following reasons: not meeting criteria by title or abstract (*n* = 175), not evaluating the outcomes of interest (*n* = 5), or not being randomized clinical trials (*n* = 8). Finally, 5 studies were included in the systematic review. The details of the selection process are illustrated in Figure 1.

### 3.2. Characteristics of the Studies Included in the Review

The included studies were RCTs conducted between 2011 and 2022 in countries in Asia (Nepal, Thailand), Europe (Spain), and North America (United States), which provides a geographically diverse perspective. All trials shared similar diagnostic criteria for acute pancreatitis, including abdominal pain, amylase or lipase elevation greater than three times the upper normal limit, and characteristic findings on cross-sectional imaging, which provides uniformity in the case definition. However, relevant differences were observed in the exclusion criteria: some studies excluded patients with comorbidities such as chronic renal failure, liver or lung disease, sepsis, or recent cardiovascular interventions, while others applied restrictions based on the time of symptom evolution or the initial care received at other centers. See Table 1.

**Figure 2:**
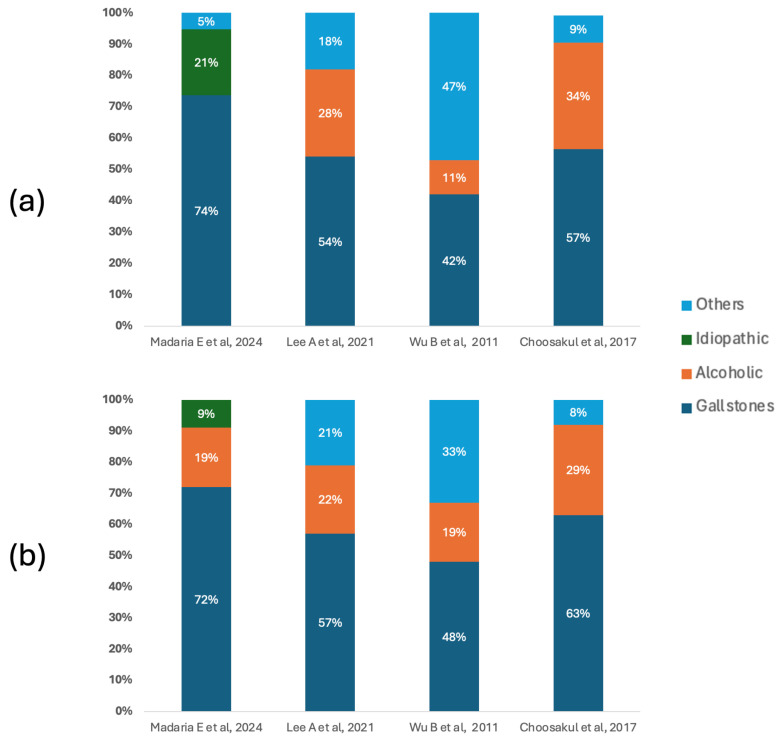
Etiology of acute pancreatitis. (**a**) Main causes of acute pancreatitis in patients who underwent fluid therapy with Lactated Ringer. (**b**) Main causes of acute pancreatitis in patients who underwent fluid therapy with Normal Saline [26,27,28,29].

Figure 2 shows the etiological distribution of acute pancreatitis in the included studies, differentiating patients according to the type of fluid therapy received. In both groups, biliary lithiasis was the predominant cause, especially in the studies by Madaria E et al. [27] and Choosakul S et al. [28], where it exceeded 70% and 60%, respectively. In the group treated with Lactated Ringer, the notable proportion of alcoholic etiologies reported by Lee A et al. [26] (28%) and Choosakul S et al. [28] (34%) stands out, while Wu B et al. [29] presented a high burden of causes classified as “other” (47%), which could indicate differences in the clinical context or inclusion criteria. In the group that received Normal Saline, the predominance of biliary lithiasis was maintained, although with a slight percentage reduction in other etiologies compared to the Ringer’s group in some studies.

### 3.3. Characteristics of the Population and the Applied Intervention

The female population ranged from 38% to 53%, with an average age of over 40 years in all studies. Despite variations in design and population, common methodological elements are identified, such as the administration of initial boluses followed by continuous infusions, with a treatment duration primarily focused on the first 72 h, except in the studies by Wu et al. [29] and Choosakul S et al. [28]. All studies applied structured hydration protocols based on body weight, with initial boluses ranging from 10 to 20 mL/kg, followed by maintenance infusions varying between 1 and 3 mL/kg/h, adjusted according to clinical response or laboratory results. Although not all studies reported the exact volume administered at 24 h, Lee et al. [26] provided quantitative data differentiated by group, highlighting a higher administration in the group treated with LR (1.75 L vs. 1.55 L in the NS group). See Table 2.

### 3.4. Results of the Risk of Bias Assessment

**Figure 3:**
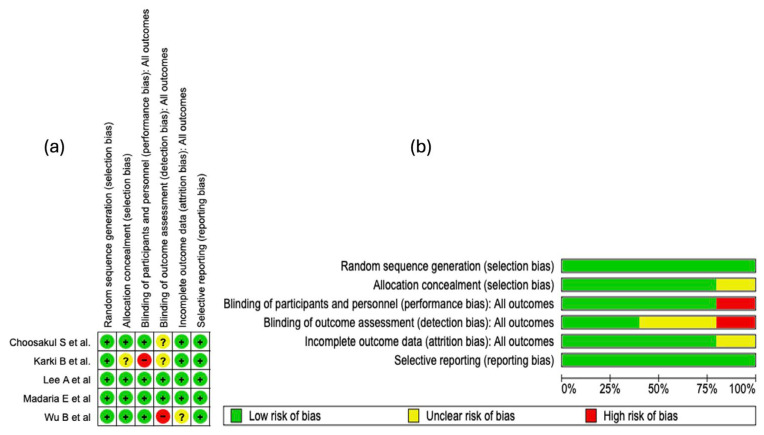
Risk of bias evaluation for the studies included in this review. (**a**) The “+” symbol denotes low risk of bias, “?” indicates unclear risk, and “−” represents high risk of bias. The colors associated with each symbol are green for low risk, yellow for unclear risk, and red for high risk. (**b**) This panel shows a summary of the identified risk of bias across all evaluated studies, displaying the percentage corresponding to each risk-of-bias item [25,26,27,28,29].

The risk of bias analysis of the included studies was carried out considering several methodological domains, as shown in Figure 3. This evaluation was performed using the RevMan 5.4^®^ tool (accessed: April 2025), which allowed for the identification of both strengths and limitations in the design and execution of the clinical trials. See Figure 3.

#### 3.4.1. Random Sequence Generation

In all of the studies incorporated into this systematic review, the generation of the random sequence was categorized as low risk of bias. This evaluation indicates that the randomization methods were described with sufficient detail and executed according to robust methodological standards. The absence of judgments reporting unclear or high risk in this domain strengthens the internal validity of the included trials and supports the reliability of the estimated effects.

#### 3.4.2. Allocation Concealment

Regarding the domain of bias due to allocation concealment, four of the five studies included in this systematic review (80%) were evaluated as “low risk of bias.” This high proportion reflects an adequate implementation and reporting of methodological strategies aimed at preventing the prediction of treatment allocation. However, in the study by

Karki B et al. [25], an unclear risk was identified in this domain due to the lack of clear information on the methods used to preserve concealment.

#### 3.4.3. Blinding of Participants and Personnel

Regarding the domain of bias due to blinding of participants and personnel, four of the five included studies were evaluated as low risk. This assessment reflects an adequate implementation of mechanisms to minimize performance bias, thus preserving neutrality in the administration of interventions and in the evolution of outcomes. In contrast, the study by Karki B et al. [25] presented a high risk in this domain, which introduces a potential threat to internal validity, given that the absence of blinding can influence the behavior of participants or clinical decisions, affecting the objectivity of the outcomes.

#### 3.4.4. Blinding of Outcome Assessment

In the domain corresponding to the blinding of outcome assessors, only two of the five included studies were evaluated as low risk of bias, which indicates an adequate implementation of mechanisms to preserve objectivity in the evaluation of results. In contrast, the studies by Karki B et al. [25] and Choosakul S et al. [28] presented an unclear risk, due to a lack of verifiable information on the protection against detection bias. Critically, the study by Wu B et al. [29] was classified as high risk, which seriously compromises internal validity, given that the absence of blinding in outcome assessment can distort the effect estimation, particularly in non-objective clinical outcomes.

#### 3.4.5. Incomplete Outcome Data

In the domain of bias due to incomplete data, four of the five included studies were classified as low risk, which suggests adequate management of losses during follow-up and sufficient transparency in the presentation of the analyzed data. However, the study by Wu B et al. [29] presented an unclear risk, due to the absence of detailed information on missing data. This prevents completely ruling out an attrition bias, particularly if the losses were not random or differentially affected the comparison groups.

#### 3.4.6. Selective Reporting

In the domain of bias due to selective reporting, all included studies were evaluated as low risk. This classification indicates an adequate correspondence between the pre-specified outcomes and those finally reported, without evidence of systematic omission of clinically relevant results. See Figure 3b.

#### 3.4.7. Summary of Risk of Bias

The methodological analysis showed that random sequence generation and selective reporting presented a low risk of bias in all of the included studies, which reflects an adequate implementation of randomization and transparency in the communication of outcomes. Regarding allocation concealment, an unclear risk was observed in approximately 20% of the studies, which could introduce vulnerability in the blind allocation of participants. Regarding the blinding of participants and personnel, a high risk of bias was identified in 20% of the studies, which represents a potential threat to the control of performance bias.

For the blinding of outcome assessors, about 40% of the studies presented an unclear risk and 20% a high risk, suggesting methodological deficiencies that could impact the objectivity of the outcome evaluation. In relation to incomplete outcome data, 20% of the studies showed an unclear risk, although the majority maintained a low risk, indicating adequate management of follow-up losses in general terms.

### 3.5. Qualitative Synthesis of the Scientific Evidence

#### 3.5.1. Mortality

Mortality was an outcome evaluated in all of the included studies [25,26,27,28,29]. Three of the five studies [25,27,28] reported a higher frequency of mortality events in the group treated with saline solution compared to the group that received Lactated Ringer. However, in each of these studies, the observed difference was marginal: the absolute number of deaths did not exceed one patient in the saline solution group, versus no deaths recorded in the Lactated Ringer group. These differences, although descriptively relevant, lack statistical significance and limit the possibility of establishing a robust association between the type of fluid administered and mortality in the analyzed contexts. In the two remaining studies [26,29], no deaths were recorded in either treatment group.

#### 3.5.2. Adverse Events

Two of the five [26,29] studies considered adverse effects among their outcomes. In the study by Wu B et al. [29], the incidence of adverse events was higher in the control group, although without reaching statistically significant differences. For their part, Lee A et al. [26] reported a higher frequency of events in the group treated with Lactated Ringer; however, this finding corresponded to a single patient, lacking clinical and statistical relevance. The low rate of events and the absence of significance in both studies limit the interpretation of real differences in terms of safety between the evaluated solutions.

### 3.6. Meta-Analysis

A meta-analysis was performed for 5 outcomes (hospital stay, admission to intensive care unit, SIRS, progression of pancreatitis, CRP) from the studies of Madaria E et al. [27], Lee A et al. [26], Karki B et al. [25], Wu B et al. [29], and Choosakul et al. [28].

#### 3.6.1. Results of the Evidence Quality Assessment

In this review, five studies were evaluated according to essential methodological criteria: randomization, blinding, description of withdrawals, adequate random allocation, and definition of selection criteria. Two studies [26,28] obtained 5 points, meeting all quality standards. Three studies [25,27,29] achieved 4 points: Karki B et al. [25], and Wu B et al. [29] did not use a double-blind design, and Madaria E et al. [27] did not adequately describe the random allocation. Despite these deficiencies, all studies maintained acceptable internal validity, ensuring the reliability of the results. See Table 3.

#### 3.6.2. Hospital Stay

**Figure 4:**
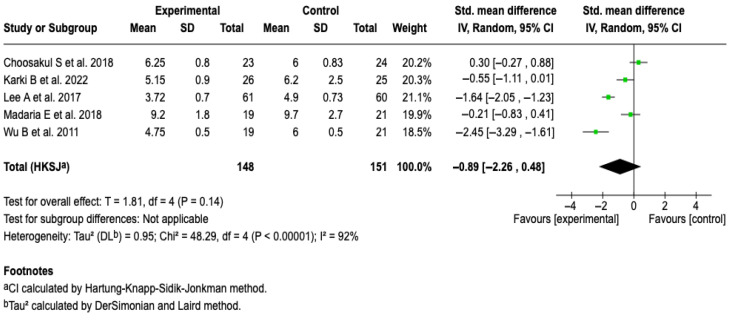
Forest plots of the effect of fluid therapy with Lactated Ringer compared to Saline Solution on hospital stay in patients with acute pancreatitis [25,26,27,28,29].

All of the studies in the review [25,26,27,28,29], in which 299 patients were included, evaluated hospital stay as an outcome. From the meta-analysis, it is evident that Lactated Ringer compared to saline solution does not reduce the hospital stay in patients with acute pancreatitis (SMD: −0.89; 95% CI: −2.26 to 0.48; *p* = 0.23). The heterogeneity test yielded a Chi^2^ value = 48.29 (*p* < 0.00001) and I^2^ = 92%, which indicates considerable heterogeneity among the included studies (See Figure 4). The subgroup analysis did not show that aggressive or moderately aggressive resuscitation was related to hospital stay (*p* = 0.23). See Figure S1. Furthermore, none of the sensitivity analyses modified the effect of the intervention on the outcome, which suggests that the results are robust. See Figures S2 and S3 of the Supplementary Material.

#### 3.6.3. Admission to Intensive Care Unit

**Figure 5:**
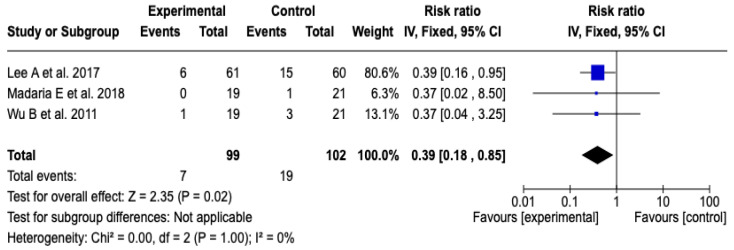
Forest plots of the effect of fluid therapy with Lactated Ringer compared to Saline Solution on ICU admission in patients with acute pancreatitis [26,27,29].

Three [26,27,29] studies with a total of 201 participants evaluated the impact of the technological intervention on the occurrence of the clinical event of interest. The meta-analysis, conducted under a fixed-effects model, showed a statistically significant reduction in the risk of ICU admission in the experimental group compared to the control group (RR: 0.39; 95% CI: 0.18 to 0.85; *p* = 0.02). No heterogeneity was observed among the included studies (Chi^2^ = 0.00; *p* = 1.00; I^2^ = 0%), which supports the consistency of the findings. See Figure 5. Likewise, the sensitivity analyses based on changing the model or removing individual studies did not show substantial changes in the magnitude or direction of the estimated effect, which reinforces the robustness of the result. See Figures S4 and S5 of the Supplementary Material.

#### 3.6.4. Progression of Pancreatitis

**Figure 6:**
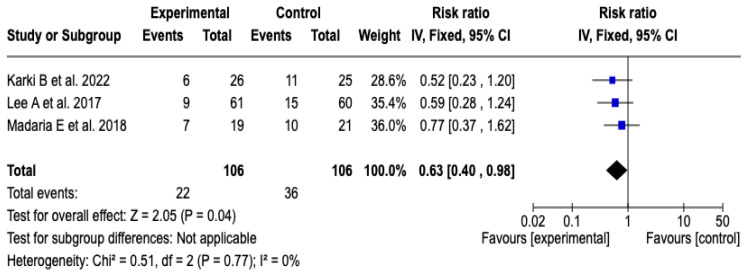
Forest plots of the effect of fluid therapy with Lactated Ringer compared to Saline Solution on the progression of pancreatitis in patients with acute pancreatitis [25,26,27].

Three [25,26,27] of the five clinical trials, which included a total of 212 participants, evaluated the impact of technological interventions on the frequency of adverse clinical events. The meta-analysis showed a reduction in the risk of progression of acute pancreatitis in the group that received Lactated Ringer (RR: 0.63; 95% CI: 0.40 to 0.98; *p* = 0.04). Heterogeneity between studies was minimal (Chi^2^ = 0.51; *p* = 0.77; I^2^ = 0%), which suggests high consistency in the estimated effects (See Figure 6). Likewise, the sensitivity analysis confirmed the stability of the results, with no relevant variations in the direction or magnitude of the effect when changing the model to random effects. See Figure S6 of the Supplementary Material.

#### 3.6.5. Development of SIRS

##### SIRS at 24 h

**Figure 7:**
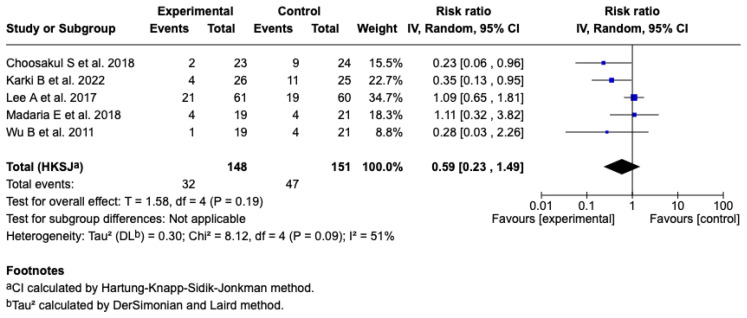
Forest plots of the effect of fluid therapy with Lactated Ringer compared to Saline Solution on the development of SIRS at 24 h in patients with acute pancreatitis [25,26,27,28,29].

Five studies [25,26,27,28,29] (*n* = 299 patients) evaluated the effect of Lactated Ringer versus saline solution on the incidence of SIRS at 24 h. The meta-analysis under a random-effects model showed a non-significant reduction in the experimental group (RR: 0.59; 95% CI: 0.23 to 1.49; *p* = 0.19), with a heterogeneity of: I^2^ = 51% (see Figure 7). The test for differences between subgroups in terms of the type of resuscitation was not significant (*p* = 0.10) (see Figure S7), and the sensitivity analyses confirmed the stability of the estimated effect. See Figures S8 and S9 of the Supplementary Material.

##### SIRS at 48 h

**Figure 8:**
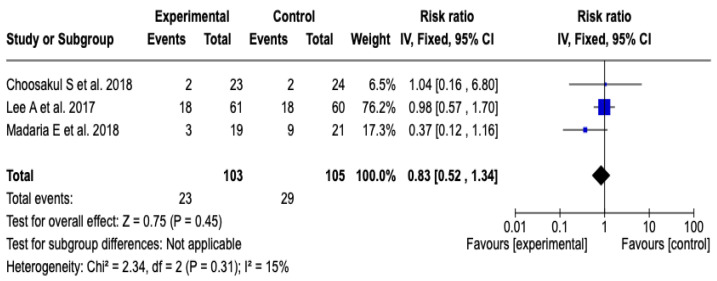
Forest plots of the effect of fluid therapy with Lactated Ringer compared to Saline Solution on the development of SIRS at 48 h in patients with acute pancreatitis [26,27,28].

Three [26,27,28] clinical trials were included that evaluated the incidence of systemic inflammatory response syndrome at 48 h after initial resuscitation with Lactated Ringer versus isotonic saline solution, with a total of 208 patients (103 in the experimental group and 105 in the control group). The meta-analysis showed low heterogeneity (Chi^2^ = 2.34; *p* = 0.31; I^2^ = 15%) and did not find a statistically significant difference between the groups (RR: 0.83; 95% CI: 0.52 to 1.34; *p* = 0.45), which indicates that there was no clear effect of the intervention on the incidence of SIRS in this period. See Figure 8. Given the low level of heterogeneity, it was not considered necessary to perform a subgroup analysis. Furthermore, a sensitivity analysis was performed applying a random-effects model, which did not modify the direction or magnitude of the estimated effect. See Figure S10 of the Supplementary Material.

##### SIRS at 72 h

**Figure 9:**
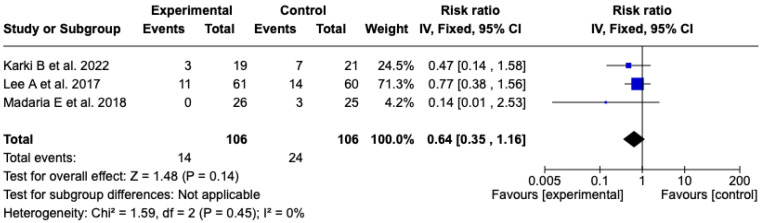
Forest plots of the effect of fluid therapy with Lactated Ringer compared to Saline Solution on the development of SIRS at 72 h in patients with acute pancreatitis [25,26,27].

The analysis included three [25,26,27] clinical trials with a total of 212 patients (106 in each group) that evaluated the incidence of SIRS at 72 h after resuscitation with Lactated Ringer or isotonic saline solution. The meta-analysis did not show a statistically significant difference between the experimental and control groups (RR: 0.64; 95% CI: 0.35 to 1.16; *p* = 0.14). Heterogeneity was null (Chi^2^ = 1.59; *p* = 0.45; I^2^ = 0%), which supports the consistency among the included studies (see Figure 9). Given the low level of heterogeneity, it was not considered pertinent to perform subgroup analyses. A sensitivity analysis was carried out using a random-effects model, which did not modify the direction or magnitude of the effect. See Figure S11 of the Supplementary Material.

#### 3.6.6. CRP Score

##### CRP at 48 h

**Figure 10:**
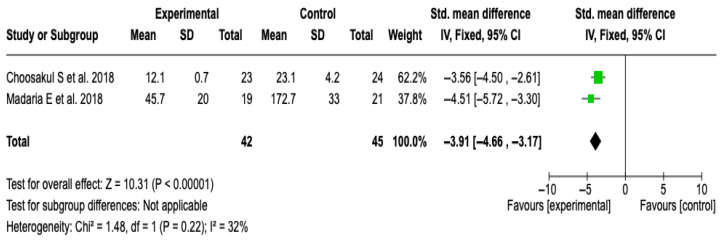
Forest plots of the effect of fluid therapy with Lactated Ringer compared to Saline Solution on the CRP score at 48 h in patients with acute pancreatitis [27,28].

Two studies [27,28] with a total of 87 patients (42 in the experimental group and 45 in the control) were included. The meta-analysis showed a significant reduction in CRP levels in favor of the group treated with Lactated Ringer (SMD: −3.91; 95% CI: −4.66 to −3.17; *p* < 0.00001), with low heterogeneity (I^2^ = 32%). See Figure 10. No subgroup analyses were performed due to the limited number of studies and the heterogeneity value. The sensitivity analysis with a random-effects model did not modify the results, which supports the robustness of the finding. See Figure S12 of the Supplementary Material.

##### CRP at 72 h

**Figure 11:**
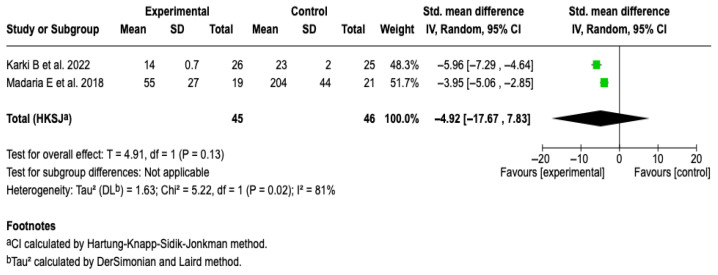
Forest plots of the effect of fluid therapy with Lactated Ringer compared to Saline Solution on the CRP score at 72 h in patients with acute pancreatitis [25,27].

Two studies [25,27] with a total of 91 patients were included. The meta-analysis under a random-effects model showed a non-significant standardized mean difference (SMD: −4.92; 95% CI: −17.67 to 7.83; *p* = 0.13). High heterogeneity was evidenced (I^2^ = 81%), with no possibility of performing subgroup analyses due to the small number of studies. See Figure 11. A sensitivity analysis was performed using an alternative model (fixed-effects model); no relevant changes were observed in the magnitude or direction of the effect. See Figure S13 of the Supplementary Material.

#### 3.6.7. Publication Bias

No formal evaluation of publication bias was performed using funnel plots or Egger’s tests, given that the number of studies included in the meta-analyses was less than 10. Below this threshold, these tools lack adequate statistical power to reliably detect asymmetry, and their application can lead to spurious results. In this scenario, any apparent asymmetry could be attributed more to random error than to a systematic publication bias, so it was considered methodologically inappropriate to apply such tests.

#### 3.6.8. Results of the GRADE Certainty of Evidence Assessment

The certainty of the evidence was evaluated using the GRADE approach. It was rated as very low for hospital stay due to problems related to risk of bias, presence of inconsistency, and imprecision. The evidence was low for most outcomes, such as ICU admission, progression of AP, SIRS at 24 h, SIRS at 72 h, and CRP at 72 h. The SIRS at 48 h outcome had moderate certainty, while CRP at 48 h was the only outcome with high certainty. The ratings reflect the overall quality of the available evidence for each result. See Table 4 and Supplementary Material (Table S1).

## 4. Discussion

### 4.1. Findings of the Review

The central focus of this systematic review and meta-analysis was to compile and analyze the results from 5 randomized clinical trials with a total of 299 patients, in order to compare the effectiveness of fluid resuscitation with Lactated Ringer versus the administration of isotonic saline solution in patients diagnosed with acute pancreatitis.

The results of the meta-analysis show that the administration of Lactated Ringer is significantly associated with a lower probability of admission to the intensive care unit (RR: 0.39; 95% CI: 0.18–0.85; *p* = 0.02), a reduction in the risk of progression of acute pancreatitis to more severe forms (RR: 0.63; 95% CI: 0.40–0.98; *p* = 0.04), and significantly lower levels of C-reactive protein at 48 h (SMD: −3.91; 95% CI: −4.66 to −3.17; *p* < 0.00001), which suggests a possible early anti-inflammatory effect. However, no statistically significant differences were observed in other clinical outcomes, such as CRP levels at 72 h (SMD: −4.92; 95% CI: −17.67 to 7.83; *p* = 0.13), the duration of hospital stay (SMD: −0.89; 95% CI: −2.26 to 0.48; *p* = 0.23), or the incidence of systemic inflammatory response syndrome at 24, 48, and 72 h after the start of fluid therapy. These findings indicate that while LR may offer early benefits in the clinical course of acute pancreatitis, additional studies are required to confirm its sustained impact over time and its effect on more robust clinical outcomes.

In addition to the quantitative results, the qualitative synthesis allowed for the incorporation of valuable information on relevant clinical outcomes that, due to the low frequency of events, could not be included in the meta-analysis. Regarding mortality, although some studies suggested a higher occurrence of deaths in the group treated with saline solution, the observed differences were minimal and lacked statistical significance, which prevents establishing a conclusive relationship between the type of fluid administered and this outcome. Similarly, adverse events were scarcely reported and without clinically significant differences between the groups, which limits the possibility of drawing solid conclusions about the comparative safety of both solutions.

### 4.2. Comparison with Previous Studies

The methodological quality of the included studies was evaluated using the Jadad scale, a tool that allowed for a rigorous discrimination of the internal validity of the clinical trials based on key methodological criteria. To address heterogeneity between studies, statistical procedures were implemented that included quantitative estimates of inconsistency and sensitivity and subgroup analyses, whenever the availability and homogeneity of the data permitted. Likewise, the certainty of the evidence for each outcome was assessed using the GRADE methodology, which enabled a structured evaluation of the degree of confidence in the results and their clinical relevance [30]. This methodological approach, based on recognized tools and standardized criteria, provides a solid foundation that strengthens the interpretive validity of the findings and overcomes the limitations of previous, less exhaustive reviews.

While some findings of the present study show agreement with previously published meta-analyses, a notable variability in the results is observed, with discrepancies between the different reviews being frequent. These differences could be attributed to the heterogeneity of the included studies, as well as to methodological variations and criteria used to evaluate the outcomes.

In this regard, a meta-analysis by Osckay et al. [17] that included 557 patients concluded that the administration of Lactated Ringer was associated with a significant reduction in the need for intensive care (RR: 0.50; 95% CI: 0.33–0.77) and in mortality, without demonstrating a significant effect on the duration of hospital stay (MD: −0.57 days, CI: −1.33–0.19). These results are consistent with the findings of the present analysis, in which a lower ICU admission rate was also observed, but without statistically significant differences in the days of hospitalization. The absence of an effect on this latter outcome could be explained by the fact that, once the systemic inflammatory response is stabilized with LR, the duration of hospitalization then depends on more complex clinical determinants, such as the appearance of local complications, nosocomial infections, or the patient’s comorbidity burden, which are not modifiable by the type of fluid therapy administered in the early phases of management [31,32]. It is worth noting as a limitation that the aforementioned meta-analysis combined data from adult and pediatric patients, which could introduce heterogeneity and affect the comparability with the results obtained in our specific population.

Despite what has been described, authors like Guzman et al. [33], while agreeing on the findings inherent to the risk of ICU admission, have documented that patients undergoing fluid therapy with LR may also have shorter hospital stays (MD: −1.10; 95% CI, −1.92 to −0.28). In addition, a recent meta-analysis by Zhao T et al. [34] reported similar benefits, observing that patients treated with LR showed lower pancreatitis severity, shorter hospital stays, and lower complication rates compared to NS.

Systemic Inflammatory Response Syndrome has been widely recognized as an early marker and predictor of poor prognosis and mortality in patients with acute pancreatitis, as it reflects the dysregulated activation of the inflammatory system that can lead to multiple organ failure [35]. However, in the present study, the administration of Lactated Ringer was not associated with a significant reduction in the incidence of SIRS at 24, 48, or 72 h after the start of treatment. This finding suggests that, although fluid therapy with LR may modulate some aspects of the inflammatory response and improve hemodynamic parameters, its specific impact on the clinical and temporal manifestation of SIRS is limited or dependent on other pathophysiological factors not directly modified by the type of intravenous solution.

The foregoing is confirmed by a meta-analysis conducted in 2022, in which after analyzing 6 studies with a total group of 549 patients, the authors found that there was no reduction in the probability of developing SIRS at 24 (OR: 0.59, 95% CI 0.22–1.62, *p* = 0.31), 48, and 72 h after treatment [36]. Similarly, Zhou et al. [37] in a meta-analysis with a smaller group of patients (*n* = 248) reported that there was no difference in the development of SIRS at 24 or 48 h or in the development of organ failure in the LR group versus the NS group. In addition to this, Aziz M et al. [38] described that the type of solution used for resuscitation of patients with AP seems to have no impact on the systemic inflammatory response syndrome (RR: 0.69, 95% CI: 0.32–1.51).

Although the available evidence suggests greater homogeneity in the results regarding the effect of fluid therapy with Lactated Ringer on the incidence of SIRS, it is essential to recognize that there is no unanimity among the studies. Some reports document significant reductions in the frequency of SIRS, as is the case of Costea et al. [6] who in their systematic review found that crystalloids, particularly Lactated Ringer, are superior to normal saline for reducing SIRS and organ failure.

CRP has been widely recognized as an inflammatory biomarker of clinical utility in monitoring the evolution of patients with acute pancreatitis [39]. Its sustained elevation correlates with the severity of the clinical picture and with the risk of local and systemic complications, so it is considered an accessible and reliable tool for prognostic stratification and evaluation of treatment response [40]. In our study, treatment with Lactated Ringer was associated with lower CRP levels at 48 h, with no significant effects at 72 h after the start of the solution, a finding that is related to what has been described in other studies. On this aspect, a meta-analysis conducted with a significant sample size of 1424 patients found that the use of Lactated Ringer leads to lower CRP levels at 48 h (SMD: −84.5; 95% CI: −40.6 to −128.4, *p* = 0.001) and 72 h (SMD: −99.2; 95% CI: −61.4 to −137, *p* = 0.001) [38]. In contrast, a previous study with more than 500 patients documented that fluid therapy with LR was associated with lower CRP levels in the studied sample (MD: −51.03; 95% CI: −231.90 to 129.84; *p* = 0.350) [17].

While our results are largely consistent with those reported by Hong et al. [41], this meta-analysis provides complementary elements. These include subgroup analyses by type of resuscitation for outcomes where it was methodologically feasible, considering the limited number of available studies, as well as the assessment of evidence certainty using GRADE. The observed differences in effect magnitude can be attributed to anticipated methodological variations, such as the type of effect measure used and the statistical model applied based on heterogeneity.

In order to ensure transparency and facilitate a detailed comparison, the details of the previous meta-analyses, including the number of studies, sample size, and main conclusions, are summarized in Table S2 of the Supplementary Material.

### 4.3. Limitations of the Studies in the Review

While the overall findings of this meta-analysis are clinically promising, it is essential to consider the methodological limitations inherent to the primary studies included, which could compromise the robustness of the results. The heterogeneity in the designs of the trials represents a significant challenge for the interpretation of the aggregated effects. Although a considerable proportion of the studies were conducted under masking conditions, others used open designs, which introduces a high risk of performance bias, as the lack of blinding could have conditioned the conduct of the clinical staff, as well as the intensity of monitoring or therapeutic decision-making [25,29]. This lack of masking also exposes the studies to detection bias, by potentially influencing the evaluation of subjective clinical outcomes [42].

Likewise, several of the included trials have limited sample sizes [27,28,29] which restricts the statistical power to detect significant differences in clinically relevant events such as mortality or the development of SIRS. Such a limitation may favor imprecise estimates or null effects due to type II error, affecting the reliability of the conclusions [43]. In this context, caution is recommended when extrapolating the results to broader populations, and the need for randomized, multicenter clinical trials that validate these findings under methodologically rigorous conditions is emphasized.

From the point of view of clinical heterogeneity, some relevant variations were observed among the included studies. Although in most of them the predominant etiology of acute pancreatitis was of biliary origin, in at least one trial a large part of the cases corresponded to pancreatitis of alcoholic cause. This etiological difference is not minor, as alcoholic pancreatitis is usually associated with different patterns of inflammation, which could modify both the clinical course and the response to initial fluid therapy. On the other hand, variability was evidenced in the inclusion criteria regarding the severity of the clinical picture. Some studies predominantly included patients with mild or moderate forms, while others contemplated severe cases from the beginning. This heterogeneity in the spectrum of severity has important implications, as it can directly influence the magnitude of the observed effect and the applicability of the results to populations with different clinical profiles.

An additional aspect that must be recognized is the inherent difficulty in ensuring the equality of the compared groups in therapeutic studies on acute pancreatitis. This condition presents marked heterogeneity in its onset, clinical course, and development of complications, which leads to a high risk of baseline imbalances between the groups, especially in studies with a limited sample size. Although this bias tends to diminish in larger cohorts, in our case, it constitutes a relevant limitation that could influence the interpretation of the results. Likewise, given that the majority of the included patients had pancreatitis of biliary origin, the extrapolation of our findings to other etiologies, particularly alcoholic pancreatitis, should be performed with caution. These considerations suggest that, rather than demonstrating a definitive difference between Lactated Ringer and saline solution, our data point to the need for larger studies with more diverse populations to establish robust conclusions.

Although we consider that our meta-analysis follows a structured and transparent methodology, certain limitations should be acknowledged, particularly regarding the characterization of potential clinical confounders. The heterogeneity among the included studies in terms of relevant comorbidities and the criteria used to assess renal function limited the possibility of conducting meaningful subgroup analyses based on these variables. For this reason, no stratified analyses were performed. Moreover, although this review focused exclusively on the comparison between crystalloid solutions for fluid resuscitation in acute pancreatitis, we recognize that a comprehensive treatment approach may involve additional therapeutic interventions that warrant further investigation.

### 4.4. Limitations of the Review

Despite the effort to conduct an exhaustive meta-analysis with valuable results, this research has limitations to consider when interpreting our results. First, although methodological standardization was maintained regarding the amount of fluid administered, our review did not delve into the intensity of fluid resuscitation. This is important to consider because it has been shown that patients who receive low-intensity resuscitation (5 to <10 mL/kg/h) are associated with more favorable overall clinical outcomes, but with a higher rate of mortality, presence of SIRS, and other complications, compared to patients who receive moderate to high-intensity fluid therapy [44].

While it was not the objective of this study, it is relevant to note that the volume of fluids administered during resuscitation is significantly associated with clinical outcomes. However, as suggested by multiple meta-analyses, the intensity of fluid therapy should be individualized according to clinical judgment, considering the specific risks of each patient [45]. In this context, although the present analysis offers essential information on the type of solution used, a more detailed characterization of the fluid resuscitation protocols could have complemented and enriched the interpretation of the results.

Additionally, this meta-analysis excluded studies that evaluated pediatric populations. While this decision was based on the criteria defined from the outset to reduce clinical heterogeneity, we recognize that the inclusion of these studies could have contributed to increasing the total sample size analyzed. Despite the potential risk of increasing heterogeneity, conducting subgroup analyses would have allowed for a stratified exploration of the intervention’s effect according to age group, partially mitigating this impact. This limitation should be considered in future updates of this evidence synthesis.

Although the C-reactive protein outcome at 48 h was rated with high certainty using the GRADE methodology, this rating was based on two randomized clinical trials. Both studies had a low risk of bias, precise estimates, consistency in the direction of the effect, and adequate clinical applicability. However, the limited size of the body of evidence represents a methodological constraint that could affect the robustness and generalizability of the findings. Therefore, this result should be interpreted with due caution.

On the other hand, the high heterogeneity observed in some results represents a non-negligible limitation, as it can compromise the certainty of the evidence and make it difficult to obtain solid conclusions. Although an attempt was made to reduce the risk of bias by exclusively including randomized clinical trials, clinical and methodological heterogeneity persisted as a factor to consider.

### 4.5. Clinical Implications

The findings of this meta-analysis suggest that Lactated Ringer is more effective than the administration of normal saline in patients with AP. It is noteworthy that the use of LR decreases the risk of ICU admission and the progression of pancreatitis. Additionally, although there was no statistical relevance in the outcomes of mortality, hospital stay, and development of SIRS, it did allow us to identify the reduction in an indirect factor associated with these outcomes and the pathophysiology of AP, such as the decrease in CRP levels at 48 h, which is a factor that reflects a lower activation of the inflammatory cascade, closely related to complications like pancreatic necrosis [40]. The use of LR as a fluid for initial fluid resuscitation has been supported by other meta-analyses, which, from a practical point of view, considerably improves critical outcomes, in addition to reducing hospital costs [17].

### 4.6. Recommendations for Future Studies

The use of LR as a fluid for initial fluid resuscitation has been supported by other meta-analyses, which, from a practical point of view, considerably improves critical outcomes, which could translate into lower hospital costs [25]. However, the current evidence is limited and heterogeneous, making it difficult to make conclusive statements regarding critical outcomes such as progression to severity or mortality. Thus, the development of future research should be focused on overcoming the limitations of the current evidence. Among them, the creation of multicenter randomized clinical trials with larger sample sizes, which offers greater external validity, and including patients in different geographical and socioeconomic contexts, improving the generalization of the results and their behavior in different populations. Furthermore, it is necessary to prioritize clinically significant outcomes, cover other possible predictors of severity and progression of AP, to determine with greater precision the most effective fluid therapy.

Finally, it is essential to explore the different strategies of low, moderate, and high-intensity fluid resuscitation, as well as consider a long-term follow-up to detect outcomes related to the recurrence of AP, functional sequelae, mortality at 30–90 days, and hospital readmissions, not only in the short term but also in the medium and long term.

## 5. Conclusions

The available evidence suggests that the administration of Lactated Ringer in patients with acute pancreatitis could offer clinical benefits, especially in reducing admission to intensive care units and the progression to severe forms of the disease. However, most of the studies are of low quality, with small samples focused mainly on biliary pancreatitis, which limits the generalization of the findings to other etiologies. In this context, the results should be interpreted with caution.

## Figures and Tables

**Table 1:** Characteristics of the studies included in the review.

Author	Year	Country	Study Type	Inclusion Criteria	Exclusion Criteria	Outcomes
Karki B et al. [25]	2022	Nepal	RCT	(1) Abdominal pain. (2) Amylase and/or lipase > 3 times the upper normal limit. (3) Cross-sectional abdominal imaging showing changes consistent with acute pancreatitis. (4) Adult patients.	(1) Symptoms for more than 48 h before coming to the hospital. (2) Being referred after initial resuscitation at another hospital. (3) Having a known history of severe cardiovascular, respiratory, renal, hepatic, hematological, or immunological disease.	Hospital stay, SIRS, progression of pancreatitis, CRP, and mortality.
Lee A et al. [26]	2021	USA	RCT	(1) Abdominal pain. (2) Amylase and/or lipase > 3 times the upper normal limit. (3) Cross-sectional abdominal imaging showing changes consistent with acute pancreatitis. (4) Adult patients.	(1) Patient with severe pancreatitis according to ATLANTA criteria. (2) History of chronic kidney disease, atrial fibrillation, liver dysfunction, pregnant, clinical signs of hypovolemia.	Hospital stay, ICU admission, SIRS, progression of pancreatitis, mortality, and adverse events.
Madaria E et al. [27]	2018	Spain	RCT	(1) Abdominal pain. (2) Amylase and/or lipase > 3 times the upper normal limit. (3) Cross-sectional abdominal imaging showing changes consistent with acute pancreatitis. (4) Adult patients.	(1) Time from onset of pain to randomization > 24 h. (2) Known history of chronic kidney disease, atrial fibrillation, chronic obstructive pulmonary disease, tuberculosis, and HIV.	Hospital stay, ICU admission, SIRS, progression of pancreatitis, CRP, and mortality.
Choosakul S et al. [28]	2018	Thailand	RCT	(1) Abdominal pain. (2) Amylase and/or lipase > 3 times the upper normal limit. (3) Characteristic findings of acute pancreatitis on tomography. (4) Adult patients.	(1) Post-ERCP acute pancreatitis. (2) NYHA class II heart failure, active myocardial ischemia. (3) Liver cirrhosis, chronic kidney disease, and cardiovascular intervention < 60 days.	Hospital stay, SIRS, CRP, and mortality.
Wu B et al. [29]	2011	USA	RCT	(1) Epigastric abdominal pain. (2) Amylase and/or lipase > 3 times the upper normal limit. (3) Confirmatory finding on cross-sectional imaging. (4) Over 18 years old.	(1) NYHA class II heart failure, active myocardial ischemia. (2) Cardiovascular intervention < 60 days before inclusion. (3) Chronic kidney disease, chronic obstructive pulmonary disease requiring home oxygen, sepsis, hypernatremia, and rhabdomyolysis.	Hospital stay, ICU admission, SIRS, progression of pancreatitis, CRP, mortality, and adverse events.

ERCP: Endoscopic Retrograde Cholangiopancreatography.

**Table 2:** Characteristics of the intervention in the studies included in the review.

Author, Year	Population	% Female	Average Age	Fluid Resuscitation Protocol	Fluids at 24 h	Treatment Duration	Conclusions
Karki B et al. [25]	*n*: 51, LR: 26, NS: 25	49	41	Initial bolus of 10 mL/kg over 60 min. Followed by an infusion of 1.5 mL/kg/h.	NR	72 h	LR showed greater efficacy than NS in decreasing SIRS, especially in the first 24 h, with additional improvements in CRP and inflammatory parameters at 72 h. There were no differences in local complications.
Lee A et al. [26]	*n:* 121, LR: 61, NS: 60	44	43	Initial bolus of 10 mL/kg over 2 h, followed by a continuous infusion of 3 mL/kg/h.	LR: 1.75 L ± 0.25, NS: 1.55 L ± 0.17	72 h	Although there was no difference in SIRS at 24 h, the use of LR in patients with AP reduced the need for intensive care and shortened hospitalization, consolidating its therapeutic benefit.
Madaria E et al. [27]	*n*: 40, LR: 19, NS: 21	53	63	Initial bolus of 10 mL/kg over 60 min, followed by an infusion of 1 mL/kg/h for 3 days.	NR	72 h	Fluid therapy with lactated ringer decreases inflammation associated with acute pancreatitis.
Choosakul S et al. [28]	*n*: 47, LR: 23, NS: 24	38	52	Initial dose of 20 mg/kg over 30 min, followed by a continuous infusion of 3.0 mg/kg/h.	NR	48 h	A greater reduction in SIRS was found in patients treated with LR compared to NS at 24 h. No differences in SIRS or mortality were evidenced at 48 h between the two types of solutions.
Wu B et al. [29]	*n*: 40, LR: 19, NS: 21	45	52	Initial dose of 20 mg/kg over 30 min, followed by a continuous infusion of 3.0 mg/kg/h.	NR	24 h	In patients with acute pancreatitis, resuscitation with Lactated Ringer was associated with less systemic inflammation compared to the use of saline during initial treatment.

NR: Not reported. LR: Lactated Ringer. NS: Normal Saline.

**Table 3:** Assessment of the quality of the evidence using the Jadad scale.

Author	Study Is Randomized	Intervention Is Double-Blind	Study Withdrawals Are Accounted for and Described	Randomization Procedure Is Adequately	Selection Criteria	Score
Karki B et al., 2022 [25]	1	0	1	1	1	4
Lee A et al., 2021 [26]	1	1	1	1	1	5
Madaria E et al., 2018 [27]	1	1	1	0	1	4
Choosakul S et al., 2018 [28]	1	1	1	1	1	5
Wu B et al., 2011 [29]	1	0	1	1	1	4

**Table 4:** GRADE certainty of evidence for the outcomes.

Outcome	Effect Size (RR, MD or SMD)	GRADE Certainty
Hospital stay	SMD: −0.89 (−2.26 to 0.48)	**⬤◯◯◯**
ICU Admission	RR: 0.39 (0.18 to 0.85)	**⬤⬤◯◯**
Progression of AP	RR: 0.63 (0.40 to 0.98)	**⬤⬤◯◯**
SIRS at 24 h	RR: 0.59 (0.23 to 1.49)	**⬤⬤◯◯**
SIRS at 48 h	RR: 0.83 (0.52 to 1.34)	**⬤⬤⬤◯**
SIRS at 72 h	RR: 0.64 (0.35 to 1.16)	**⬤⬤◯◯**
CRP at 48 h	SMD: −3.91 (−4.66 to −3.17)	**⬤⬤⬤⬤**
CRP at 72 h	SMD: −4.92 (−17.67 to 7.83)	**⬤⬤◯◯**

⬤**◯◯◯**: Very low ⬤⬤**◯◯**: Low; ⬤⬤⬤**◯**: Moderate; ⬤⬤⬤⬤: High.

## Data Availability

The raw data supporting the conclusions of this article are available in the published studies included in the meta-analysis and can be accessed through the respective publications.

## Supplementary Materials

The following supporting information can be downloaded at https://www.mdpi.com/article/10.3390/diseases13090300/s1. Figure S1: Comparison of the effect of Ringer’s lactate and normal saline on hospital length of stay in acute pancreatitis according to the type of fluid resuscitation; Figure S2: Sensitivity analysis: comparison of the effect of Ringer’s lactate and normal saline on hospital length of stay in acute pancreatitis, modifying the meta-analysis model; Figure S3: Sensitivity analysis: comparison of the effect of Ringer’s lactate and normal saline on hospital length of stay in acute pancreatitis, excluding the study by Wu B et al. [29]; Figure S4: Sensitivity analysis of the effect of Ringer’s lactate versus normal saline on ICU admission in patients with acute pancreatitis, modifying the meta-analysis model; Figure S5: Sensitivity analysis of the effect of Ringer’s lactate versus normal saline on ICU admission in patients with acute pancreatitis, excluding the study by Wu B et al. [29]; Figure S6: Sensitivity analysis of the effect of Ringer’s lactate versus normal saline on progression of acute pancreatitis, modifying the meta-analysis model; Figure S7: Effect of Ringer’s lactate versus normal saline on SIRS at 24 h in patients with acute pancreatitis: subgroup analysis according to the type of fluid resuscitation; Figure S8: Sensitivity analysis of the effect of Ringer’s lactate versus normal saline on SIRS at 24 h in patients with acute pancreatitis, modifying the meta-analysis model; Figure S9: Sensitivity analysis of the effect of Ringer’s lactate versus normal saline on SIRS at 24 h in acute pancreatitis, excluding the study by Wu B et al. [29]; Figure S10: Sensitivity analysis of the effect of Ringer’s lactate versus normal saline on SIRS at 48 h in patients with acute pancreatitis, modifying the meta-analysis model; Figure S11: Sensitivity analysis of the effect of Ringer’s lactate versus normal saline on SIRS at 72 h in patients with acute pancreatitis, modifying the meta-analysis model; Figure S12: Sensitivity analysis of the effect of Ringer’s lactate versus normal saline on 48 h CRP levels in acute pancreatitis, modifying the meta-analytic model; Figure S13: Sensitivity analysis of the effect of Ringer’s lactate versus normal saline on 72 h CRP levels in acute pancreatitis, modifying the meta-analytic model. Table S1. Summary of Findings for the Primary Outcomes of the Meta-analysis. Table S2. Summary of meta-analyses on Ringer’s lactate versus normal saline in patients with acute pancreatitis.
