# Supplementary material for: Fluid Resuscitation with Lactated Ringer vs. Normal Saline in Acute Pancreatitis: A Systematic Review and Meta-Analysis of Clinical Trials
Source: Diseases. 2025 Sep 10;13(9):300. doi: 10.3390/diseases13090300 (PMC12468465; doi:10.3390/diseases13090300)
Supplement: Supplementary file 1 [file diseases-13-00300-s001.zip › Tabla S1. SOF.pdf]

Author(s): Freiser Eceomo Cruz Mosquera, Elizabeth Camacho Benítez, Mariatta Catalina Ceballo Benavides, Julián Esteban Castillo Muñoz, Carlos Andrés Castañeda and Yamil Liscano
Question: Fluid Resuscitation with Lactated Ringer’s vs. Normal Saline in Acute Pancreatitis:
Setting:
Bibliography: Karki, B.; Thapa, S.; Khadka, D.; Karki, S.; Shrestha, R.; Khanal, A.; Shrestha, R.; Paudel, B.N. Intravenous Ringers Lactate versus Normal Saline for Predominantly Mild Acute Pancreatitis in a Nepalese Tertiary Hospital. PLoS ONE 2022, 17, e0263221, doi:10.1371/journal.pone.0263221.Lee, A.; Ko, C.; Buitrago, C.; Hiramoto, B.; Hilson, L.; Buxbaum, J.; Lee, A.; Ko, C.; Buitrago, C.; Hiramoto, B.; et al. Lactated Ringers vs Normal Saline Resuscitation for Mild Acute Pancreatitis: A Randomized Trial. Gastroenterology 2021, 160, 955-957.e4, doi:10.1053/j.gastro.2020.10.044.de-Madaria, E.; Herrera-Marante, I.; González-Camacho, V.; Bonjoch, L.; Quesada-Vázquez, N.; Almenta-Saavedra, I.; Miralles-Maciá, C.; Acevedo-Piedra, N.G.; Roger-Ibáñez, M.; Sánchez-Marin, C.; et al. Fluid Resuscitation with Lactated Ringer’s Solution vs Normal Saline in Acute Pancreatitis: A Triple-Blind, Randomized, Controlled Trial. United European Gastroenterology Journal 2018, 6, 63–72, doi:10.1177/2050640617707864.Choosakul, S.; Harinwan, K.; Chirapongsathorn, S.; Opuchar, K.; Sanpajit, T.; Piyanirun, W.; Puttapitakpong, C. Comparison of Normal Saline versus Lactated Ringer’s Solution for Fluid Resuscitation in Patients with Mild Acute Pancreatitis, A Randomized Controlled Trial. Pancreatology 2018, 18, 507–512, doi:10.1016/j.pan.2018.04.016.Wu, B.U.; Hwang, J.Q.; Gardner, T.H.; Repas, K.; Delee, R.; Yu, S.; Smith, B.; Banks, P.A.; Conwell, D.L. Lactated Ringer’s Solution Reduces Systemic Inflammation Compared With Saline in Patients With Acute Pancreatitis. Clinical Gastroenterology and Hepatology 2011, 9, 710-717.e1, doi:10.1016/j.cgh.2011.04.026

Certainty assessment							№ of patients		Effect		Certainty	Importance
**№ of studies**	**Study design**	**Risk of bias**	**Inconsistency**	**Indirectness**	**Imprecision**	**Other considerations**	**Ringer Lactate**	**saline solution**	**Relative (95% CI)**	**Absolute (95% CI)**		
**Hospital stay**												
5	randomised trials	serious[a]	serious[b]	not serious[c]	serious[c]	none	148	151	-	**0** (0 to 0)	⊕◯◯◯ Very low[a,b,c]	
**Admission to intensive care unit**												
3	randomised trials	serious[d]	not serious	not serious	serious[e]	none	7/99 (7.1%)	19/102 (18.6%)	**RR 0.39** (0.18 to 0.85)	**114 fewer per 1,000** (from 153 fewer to 28 fewer)	⊕⊕◯◯ Low[d,e]	
**Acute pancreatitis progression**												
3	randomised trials	serious[f]	not serious	not serious	serious[g]	none	22/106 (20.8%)	36/106 (34.0%)	**RR 0.63** (0.40 to 0.98)	**126 fewer per 1,000** (from 204 fewer to 7 fewer)	⊕⊕◯◯ Low[f,g]	
**SIRS at 24 hours**												
5	randomised trials	serious[a]	not serious	not serious	serious[h]	none	32/148 (21.6%)	47/151 (31.1%)	**RR 0.59** (0.23 to 1.49)	**128 fewer per 1,000** (from 240 fewer to 153 more)	⊕⊕◯◯ Low[a,h]	
								0.0%		**0 fewer per 1,000** (from 0 fewer to 0 fewer)		
**SIRS at 48 hours**												
3	randomised trials	not serious	not serious	not serious	serious[i]	none	23/103 (22.3%)	29/105 (27.6%)	**RR 0.83** (0.52 to 1.34)	**47 fewer per 1,000** (from 133 fewer to 94 more)	⊕⊕⊕◯ Moderate[i]	
**SIRS at 72 hours**												
2	randomised trials	serious[f]	not serious	not serious	serious[i]	none	14/106 (13.2%)	24/106 (22.6%)	**RR 0.64** (0.35 to 1.16)	**82 fewer per 1,000** (from 147 fewer to 36 more)	⊕⊕◯◯ Low[f,i]	
**PCR at 48**												
2	randomised trials	not serious	not serious	not serious	not serious	none	42	45	-	**0** (0 to 0)	⊕⊕⊕⊕ High	
**PCR at 72 hours**												
2	randomised trials	serious[j]	serious[k]	not serious	not serious	none	0	0	-	**0** (0 to 0)	⊕⊕◯◯ Low[j,k]	

CI: confidence interval; **RR:** risk ratio

Explanations

a. Although most studies are low risk, the presence of high risk in critical domains (such as blinding) in 40% of the included studies, combined with uncertain assessments in key aspects of selection and data loss, may affect confidence in the results.
b. The meta-analysis showed substantial heterogeneity, with an I^2 value of 92%, which indicates considerable variability in effect estimates across studies that is unlikely to be due to chance alone. Although the direction of effect was generally consistent across studies, the magnitude varied considerably, and subgroup analyses were insufficient to fully explain this heterogeneity. Therefore, we downgraded the certainty of evidence by one level due to serious inconsistency.

c. The overall standard mean difference (SMD) was –0.89 with a 95% confidence interval ranging from –2.26 to 0.48. This wide confidence interval crosses the line of no effect (0) and spans both potentially important benefit and harm, indicating serious imprecision. Additionally, the total sample size (n=299) is relatively small and does not meet the optimal information size (OIS) for detecting a precise estimate. Therefore, the certainty of evidence was downgraded by one level due to imprecision.
d. The risk of bias is lowered given that the Wu B study represents 33% of the total number of studies and its effect significantly influences the overall estimate, its potentially biased impact cannot be ignored.
e. The width of the 95% confidence intervals (CIs) across the included studies indicates substantial imprecision. Although point estimates are consistent (approximately OR 0.37–0.39), the CIs are wide and cross key clinical decision thresholds. Specifically, Madaira study shows a 95% CI of 0.02 to 8.50, encompassing both a very large benefit and potential harm, while Wu's study (95% CI: 0.18 to 3.25) includes both a moderate reduction in risk and no effect. This degree of uncertainty around the effect estimate means the evidence is not sufficiently precise to support a strong recommendation, warranting a downgrade of one level for imprecision.
f. Among the three included studies, two were judged to have low overall risk of bias, while one study presented a high overall risk of bias, potentially affecting the credibility of its results. Given that this study contributes meaningfully to the pooled estimate, the certainty of the evidence was downgraded by one level.
g. : All three included studies report wide 95% confidence intervals that cross the line of no effect (RR = 1.0) and encompass both a moderate reduction in risk and no clinically important effect. For instance, the confidence intervals range from 0.23 to 1.20, 0.28 to 1.24, and 0.37 to 1.62. These intervals indicate a lack of statistical significance and substantial uncertainty around the true effect size
h. Of the five studies included, three reported 95% confidence intervals that crossed the line of no effect, indicating uncertainty about whether the intervention is beneficial. Only two studies showed statistically significant results with confidence intervals that did not include the null value. This distribution reflects inconsistency in the statistical significance across the body of evidence, suggesting a lack of precision in the pooled estimate.
i. All three studies reported 95% confidence intervals that include the null value (RR = 1.0), indicating that the true effect could range from no effect to a meaningful benefit or harm. This reflects substantial uncertainty about the actual impact of the intervention, as the possibility of no effect cannot be ruled out.
j. Of the two included studies, one was assessed as having low risk of bias and the other as having high risk of bias. The study with high risk of bias could distort the overall effect estimate due to issues such as selective reporting or inadequate allocation concealment. Given that the study with high risk of bias contributes significantly to the pooled effect of the meta-analysis, the certainty of the evidence is downgraded by one level in accordance with GRADE recommendations
k. The meta-analysis showed substantial heterogeneity, with an I^2 value of 92%, which indicates considerable variability in effect estimates across studies that is unlikely to be due to chance alone. Although the direction of effect was generally consistent across studies, the magnitude varied considerably, and subgroup analyses were insufficient to fully explain this heterogeneity. Therefore, we downgraded the certainty of evidence by one level due to serious inconsistency.
