# Supplementary material for: Fluid Resuscitation with Lactated Ringer vs. Normal Saline in Acute Pancreatitis: A Systematic Review and Meta-Analysis of Clinical Trials
Source: Diseases. 2025 Sep 10;13(9):300. doi: 10.3390/diseases13090300 (PMC12468465; doi:10.3390/diseases13090300)
Supplement: Supplementary file 1 [file diseases-13-00300-s001.zip › Tabla S2.pdf]

Table S2. Summary of meta-analyses on Ringer's lactate versus normal saline in patients with acute pancreatitis.

Author, year	Number of studies	Types of studies included	Population	Sample size	Type of fluids	Conclusions
Di Martino et al, 2021[13]	15	Randomized clinical trials	Adults	n: 1703	Ringer's lactate, Normal saline, Hydroxyethyl starch	The use of Ringer's lactate is associated with a lower incidence of serious adverse events compared with normal saline.
Ocskay K et al, 2023[17]	8	Randomized clinical trials	Adults and pediatric patients	n: 557	Ringer's lactate, Normal saline	Ringer's lactate is associated with a lower risk of severe pancreatitis, mortality, ICU admission, organ failure, and local complications compared with normal saline.
Guzmán-Calderón E et al, 2022[33]	4	Randomized clinical trials	Adults	n: 248	Ringer's lactate, Normal saline	Patients treated with Ringer's lactate had shorter hospital stays and a lower risk of ICU admission compared with the normal saline group.
Zhao T et al, 2025[34]	10	Randomized clinical trials and observational studies	Adults	n: 1500	Ringer's lactate, Normal saline	Compared with normal saline, patients treated with Ringer's lactate had lower risk of moderate-to-severe acute pancreatitis, shorter hospital stays, fewer ICU admissions, and fewer local complications.
Vedantam, S et al, 2022[36]	6	Randomized clinical trials and observational studies	Adults	n: 549	Ringer's lactate, Normal saline	No differences were observed between Ringer's lactate and normal saline in the development of systemic inflammatory response syndrome at 24, 48, and 72 hours, mortality, or other secondary outcomes; however, Ringer's lactate was associated with less need for ICU admission.
Zhou S et al, 2021[37]	4	Randomized clinical trials	Adults	n: 248	Ringer's lactate, Normal saline	Patients resuscitated with Ringer's lactate had lower risk of moderately severe or severe pancreatitis, ICU admission, and local complications.
Aziz M et al, 2021[38]	6	Randomized clinical trials and observational studies	Adults	n: 549	Ringer's lactate, Normal saline	No significant differences were found in mortality or systemic inflammatory response syndrome at 24 hours between Ringer's lactate and normal saline; however, Ringer's lactate was associated with fewer ICU admissions.

Hong J et al, 2025[42]	9	Randomized clinical trials and observational studies	Adults	n: 1424	Ringer's lactate, Normal saline	Ringer's lactate was associated with lower risk of moderately severe or severe pancreatitis, fewer ICU admissions, fewer local complications, lower C-reactive protein levels, and shorter hospital stays compared with normal saline.
Iqbal Q et al, 2018	5	Randomized clinical trials and observational studies	Adults	n: 428	Ringer's lactate, Normal saline	Ringer's lactate was associated with lower risk of developing systemic inflammatory response syndrome at 24 hours, while the reduction in mortality showed a trend but was not statistically significant.
