# Supplementary material for: Fluid Resuscitation with Lactated Ringer vs. Normal Saline in Acute Pancreatitis: A Systematic Review and Meta-Analysis of Clinical Trials
Source: Diseases. 2025 Sep 10;13(9):300. doi: 10.3390/diseases13090300 (PMC12468465; doi:10.3390/diseases13090300)

Figure S1. Comparison of the effect of Ringer's lactate and normal saline on hospital length of stay in acute pancreatitis according to the type of fluid resuscitation.

Study or Subgroup	Experimental Mean	Experimental SD	Experimental Total	Control Mean	Control SD	Control Total	Weight	Std. mean difference IV, Random, 95% CI
**1.1.1 Moderately aggressive fluid therapy**								
Karki B et al. 2022	5.15	0.9	26	6.2	2.5	25	20.3%	-0.55 [-1.11 , 0.01]
Lee A et al. 2017	3.72	0.7	61	4.9	0.73	60	21.1%	-1.64 [-2.05 , -1.23]
Madaria E et al. 2018	9.2	1.8	19	9.7	2.7	21	19.9%	-0.21 [-0.83 , 0.41]
**Subtotal (HKSJ[a])**			**106**			**106**	**61.3%**	**-0.82 [-2.69 , 1.04]**
Test for overall effect: T = 1.90, df = 2 (P = 0.20)								
Heterogeneity: Tau² (DL[b]) = 0.57; Chi² = 17.76, df = 2 (P = 0.0001); I² = 89%								
**1.1.2 Aggressive fluid therapy**								
Choosakul S et al. 2018	6.25	0.8	23	6	0.83	24	20.2%	0.30 [-0.27 , 0.88]
Wu B et al. 2011	4.75	0.5	19	6	0.5	21	18.5%	-2.45 [-3.29 , -1.61]
**Subtotal (HKSJ[a])**			**42**			**45**	**38.7%**	**-1.06 [-18.54 , 16.42]**
Test for overall effect: T = 0.77, df = 1 (P = 0.58)								
Heterogeneity: Tau² (DL[b]) = 3.65; Chi² = 28.08, df = 1 (P < 0.00001); I² = 96%								
**Total (HKSJ[a])**			**148**			**151**	**100.0%**	**-0.89 [-2.26 , 0.48]**

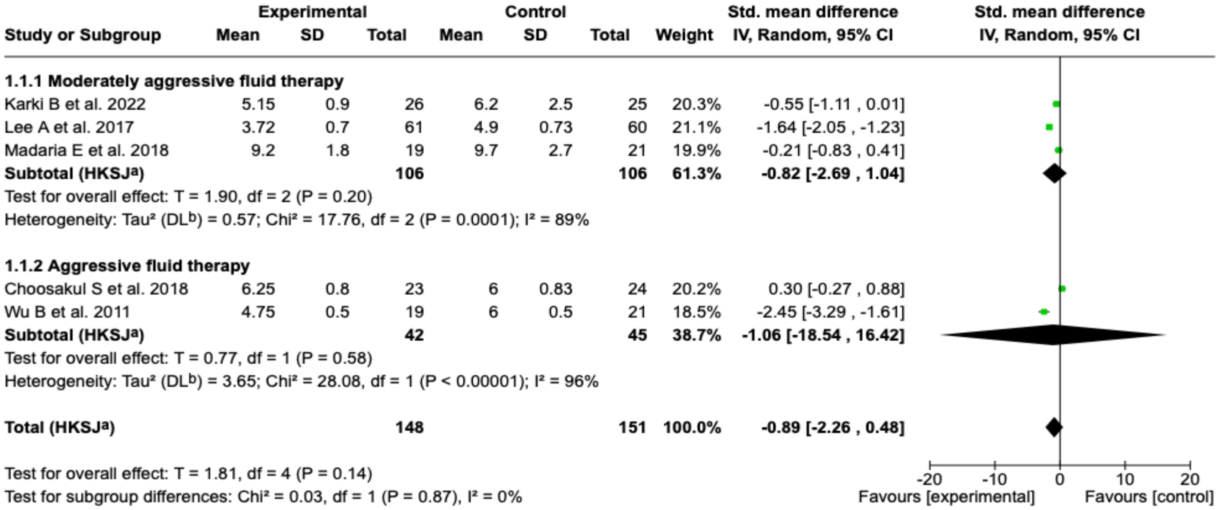

Test for overall effect: T = 1.81, df = 4 (P = 0.14)
Test for subgroup differences: Chi² = 0.03, df = 1 (P = 0.87), I² = 0%
Heterogeneity: Tau² (DL[b]) = 0.95; Chi² = 48.29, df = 4 (P < 0.00001); I² = 92%

Footnotes
[a]CI calculated by Hartung-Knapp-Sidik-Jonkman method.
[b]Tau² calculated by DerSimonian and Laird method.

Figure S2. Sensitivity analysis: comparison of the effect of Ringer's lactate and normal saline on hospital length of stay in acute pancreatitis, modifying the meta-analysis model.

Study or Subgroup	Experimental Mean	Experimental SD	Experimental Total	Control Mean	Control SD	Control Total	Weight	Std. mean difference IV, Random, 95% CI
Choosakul S et al. 2018	6.25	0.8	23	6	0.83	24	24.7%	0.30 [-0.27 , 0.88]
Karki B et al. 2022	5.15	0.9	26	6.2	2.5	25	24.9%	-0.55 [-1.11 , 0.01]
Lee A et al. 2017	3.72	0.7	61	4.9	0.73	60	26.2%	-1.64 [-2.05 , -1.23]
Madaria E et al. 2018	9.2	1.8	19	9.7	2.7	21	24.2%	-0.21 [-0.83 , 0.41]
**Total (Wald[a])**			**129**			**130**	**100.0%**	**-0.54 [-1.37 , 0.28]**

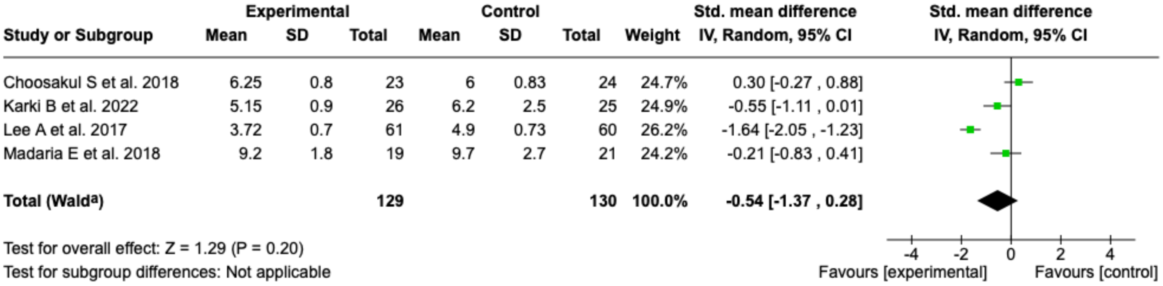

Test for overall effect: Z = 1.29 (P = 0.20)
Test for subgroup differences: Not applicable
Heterogeneity: Tau² (REML[b]) = 0.63; Chi² = 33.94, df = 3 (P < 0.00001); I² = 89%

Footnotes
[a]CI calculated by Wald-type method.
[b]Tau² calculated by Restricted Maximum-Likelihood method.

Figure S3. Sensitivity analysis: comparison of the effect of Ringer's lactate and normal saline on hospital length of stay in acute pancreatitis, excluding the study by Wu B et al. [35]

Study or Subgroup	Experimental Mean	Experimental SD	Experimental Total	Control Mean	Control SD	Control Total	Weight	Std. mean difference IV, Fixed, 95% CI
Choosakul S et al. 2018	6.25	0.8	23	6	0.83	24	18.8%	0.30 [-0.27 , 0.88]
Karki B et al. 2022	5.15	0.9	26	6.2	2.5	25	19.8%	-0.55 [-1.11 , 0.01]
Lee A et al. 2017	3.72	0.7	61	4.9	0.73	60	36.4%	-1.64 [-2.05 , -1.23]
Madaria E et al. 2018	9.2	1.8	19	9.7	2.7	21	16.1%	-0.21 [-0.83 , 0.41]
Wu B et al. 2011	4.75	0.5	19	6	0.5	21	8.8%	-2.45 [-3.29 , -1.61]
**Total**			**148**			**151**	**100.0%**	**-0.90 [-1.15 , -0.65]**

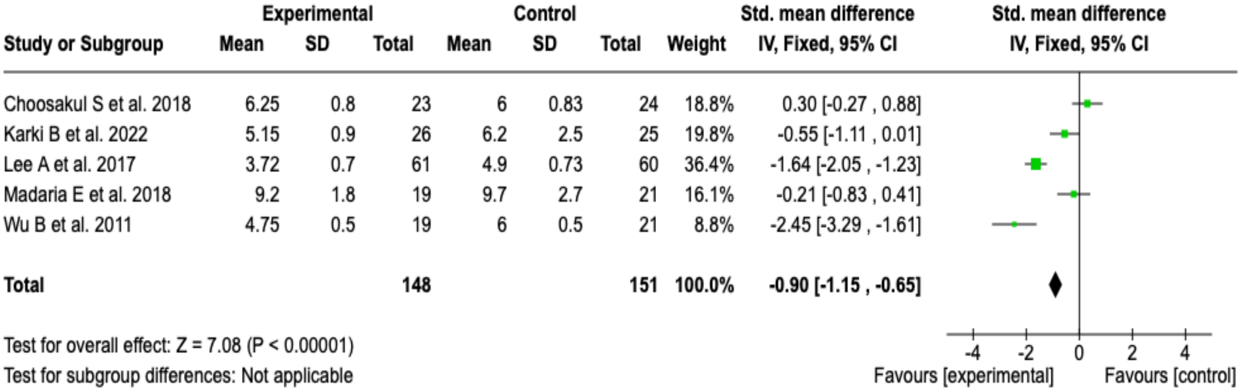

Test for overall effect: Z = 7.08 (P < 0.00001)
Test for subgroup differences: Not applicable
Heterogeneity: Chi² = 48.29, df = 4 (P < 0.00001); I² = 92%

Figure S4. Sensitivity analysis of the effect of Ringer's lactate versus normal saline on ICU admission in patients with acute pancreatitis, modifying the meta-analysis model

Study or Subgroup	Experimental Events	Experimental Total	Control Events	Control Total	Weight	Risk ratio IV, Random, 95% CI
Lee A et al. 2017	6	61	15	60	80.6%	0.39 [0.16 , 0.95]
Madaria E et al. 2018	0	19	1	21	6.3%	0.37 [0.02 , 8.50]
Wu B et al. 2011	1	19	3	21	13.1%	0.37 [0.04 , 3.25]
**Total (HKSJ[a])**		**99**		**102**	**100.0%**	**0.39 [0.36 , 0.42]**
Total events:	7		19			

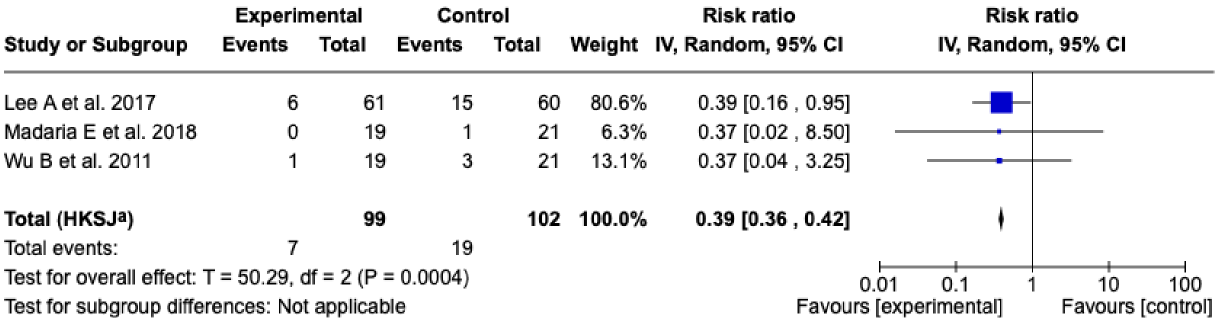

Test for overall effect: T = 50.29, df = 2 (P = 0.0004)
Test for subgroup differences: Not applicable
Heterogeneity: Tau^{2} (DL[b]) = 0.00; Chi^{2} = 0.00, df = 2 (P = 1.00); I^{2} = 0%

Footnotes
[a]CI calculated by Hartung-Knapp-Sidik-Jonkman method.
[b]Tau^{2} calculated by DerSimonian and Laird method.

Figure S5. Sensitivity analysis of the effect of Ringer's lactate versus normal saline on ICU admission in patients with acute pancreatitis, excluding the study by Wu B et al. [35]

Study or Subgroup	Experimental Events	Experimental Total	Control Events	Control Total	Weight	Risk ratio IV, Fixed, 95% CI
Lee A et al. 2017	6	61	15	60	92.8%	0.39 [0.16 , 0.95]
Madaria E et al. 2018	0	19	1	21	7.2%	0.37 [0.02 , 8.50]
**Total**		**80**		**81**	**100.0%**	**0.39 [0.17 , 0.91]**
Total events:	6		16			

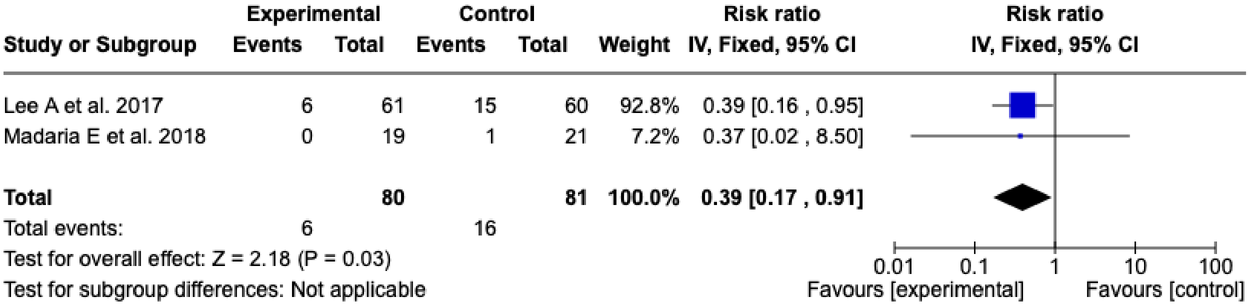

Test for overall effect: Z = 2.18 (P = 0.03)
Test for subgroup differences: Not applicable
Heterogeneity: Chi^{2} = 0.00, df = 1 (P = 0.97); I^{2} = 0%

Figure S6. Sensitivity analysis of the effect of Ringer's lactate versus normal saline on progression of acute pancreatitis, modifying the meta-analysis model.

Study or Subgroup	Experimental Events	Experimental Total	Control Events	Control Total	Weight	Risk ratio IV, Random, 95% CI
Karki B et al. 2022	6	26	11	25	28.6%	0.52 [0.23 , 1.20]
Lee A et al. 2017	9	61	15	60	35.4%	0.59 [0.28 , 1.24]
Madaria E et al. 2018	7	19	10	21	36.0%	0.77 [0.37 , 1.62]
**Total (HKSJ[a])**		**106**		**106**	**100.0%**	**0.63 [0.38 , 1.03]**
Total events:	22		36			

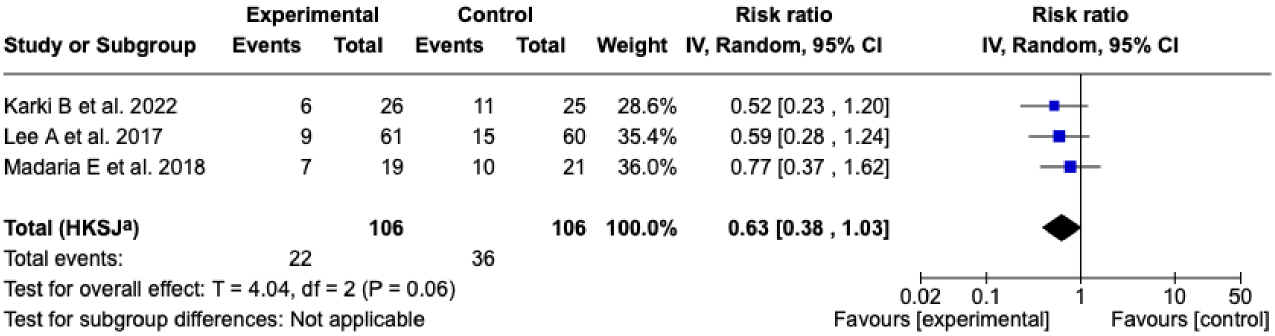

Test for overall effect: T = 4.04, df = 2 (P = 0.06)
Test for subgroup differences: Not applicable
Heterogeneity: Tau^{2} (DL[b]) = 0.00; Chi^{2} = 0.51, df = 2 (P = 0.77); I^{2} = 0%

Footnotes
[a]CI calculated by Hartung-Knapp-Sidik-Jonkman method.
[b]Tau^{2} calculated by DerSimonian and Laird method.

Figure S7. Effect of Ringer's lactate versus normal saline on SIRS at 24 hours in patients with acute pancreatitis: subgroup analysis according to the type of fluid resuscitation.

	Experimental		Control			Risk ratio
Study or Subgroup	Events	Total	Events	Total	Weight	IV, Random, 95% CI
**4.1.1 Moderately aggressive fluid therapy**						
Karki B et al. 2022	4	26	11	25	22.7%	0.35 [0.13 , 0.95]
Lee A et al. 2017	21	61	19	60	34.7%	1.09 [0.65 , 1.81]
Madaria E et al. 2018	4	19	4	21	18.3%	1.11 [0.32 , 3.82]
**Subtotal (HKSJ[a])**		**106**		**106**	**75.7%**	**0.79 [0.16 , 3.78]**
Total events:	29		34			
Test for overall effect: T = 0.65, df = 2 (P = 0.58)						
Heterogeneity: Tau² (DL[b]) = 0.21; Chi² = 4.04, df = 2 (P = 0.13); I² = 50%						
**4.1.2 Aggressive fluid therapy**						
Choosakul S et al. 2018	2	23	9	24	15.5%	0.23 [0.06 , 0.96]
Wu B et al. 2011	1	19	4	21	8.8%	0.28 [0.03 , 2.26]
**Subtotal (HKSJ[a])**		**42**		**45**	**24.3%**	**0.25 [0.09 , 0.69]**
Total events:	3		13			
Test for overall effect: T = 17.29, df = 1 (P = 0.04)						
Heterogeneity: Tau² (DL[b]) = 0.00; Chi² = 0.02, df = 1 (P = 0.89); I² = 0%						
**Total (HKSJ[a])**		**148**		**151**	**100.0%**	**0.59 [0.23 , 1.49]**
Total events:	32		47			
Test for overall effect: T = 1.58, df = 4 (P = 0.19)						
Test for subgroup differences: Chi² = 2.75, df = 1 (P = 0.10), I² = 63.6%						
Heterogeneity: Tau² (DL[b]) = 0.30; Chi² = 8.12, df = 4 (P = 0.09); I² = 51%						

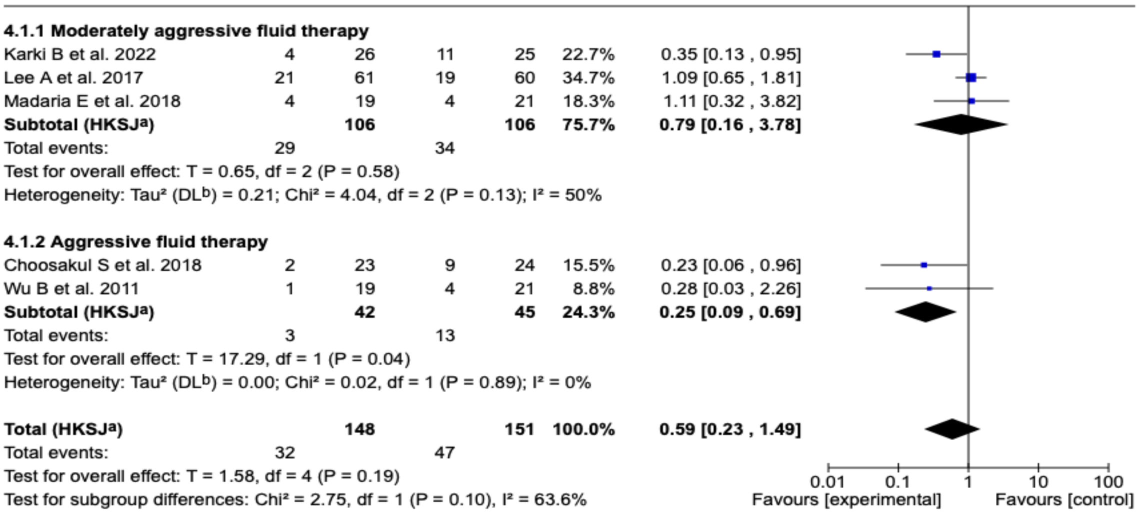

Footnotes

[a]CI calculated by Hartung-Knapp-Sidik-Jonkman method.

[b]Tau² calculated by DerSimonian and Laird method.

Figure S8. Sensitivity analysis of the effect of Ringer's lactate versus normal saline on SIRS at 24 hours in patients with acute pancreatitis, modifying the meta-analysis model.

	Experimental		Control			Risk ratio
Study or Subgroup	Events	Total	Events	Total	Weight	IV, Fixed, 95% CI
Choosakul S et al. 2018	2	23	9	24	7.9%	0.23 [0.06 , 0.96]
Karki B et al. 2022	4	26	11	25	15.9%	0.35 [0.13 , 0.95]
Lee A et al. 2017	21	61	19	60	62.1%	1.09 [0.65 , 1.81]
Madaria E et al. 2018	4	19	4	21	10.4%	1.11 [0.32 , 3.82]
Wu B et al. 2011	1	19	4	21	3.6%	0.28 [0.03 , 2.26]
**Total**		**148**		**151**	**100.0%**	**0.77 [0.51 , 1.14]**
Total events:	32		47			
Test for overall effect: Z = 1.31 (P = 0.19)						
Test for subgroup differences: Not applicable						
Heterogeneity: Chi² = 8.12, df = 4 (P = 0.09); I² = 51%						

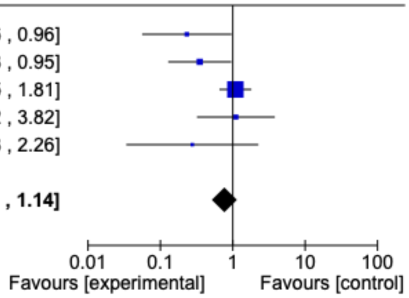

Figure S9. Sensitivity analysis of the effect of Ringer's lactate versus normal saline on SIRS at 24-hour in acute pancreatitis, excluding the study by Wu B et al. [35]

	Experimental		Control			Risk ratio
Study or Subgroup	Events	Total	Events	Total	Weight	IV, Random, 95% CI
Choosakul S et al. 2018	2	23	9	24	17.4%	0.23 [0.06 , 0.96]
Karki B et al. 2022	4	26	11	25	25.1%	0.35 [0.13 , 0.95]
Lee A et al. 2017	21	61	19	60	37.2%	1.09 [0.65 , 1.81]
Madaria E et al. 2018	4	19	4	21	20.4%	1.11 [0.32 , 3.82]
**Total (HKSJ[a])**		**129**		**130**	**100.0%**	**0.63 [0.19 , 2.11]**
Total events:	31		43			
Test for overall effect: T = 1.22, df = 3 (P = 0.31)						
Test for subgroup differences: Not applicable						
Heterogeneity: Tau² (DL[b]) = 0.34; Chi² = 7.19, df = 3 (P = 0.07); I² = 58%						

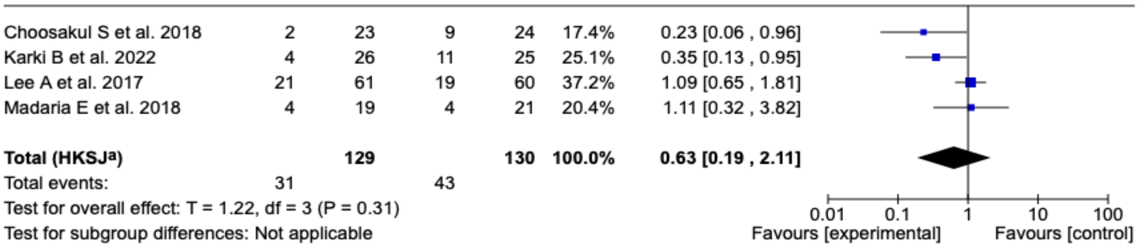

Footnotes

[a]CI calculated by Hartung-Knapp-Sidik-Jonkman method.

[b]Tau² calculated by DerSimonian and Laird method.

Figure S10. Sensitivity analysis of the effect of Ringer's lactate versus normal saline on SIRS at 48 hours in patients with acute pancreatitis, modifying the meta-analysis model.

Study or Subgroup	Experimental Events	Experimental Total	Control Events	Control Total	Weight	Risk ratio IV, Random, 95% CI
Choosakul S et al. 2018	2	23	2	24	9.2%	1.04 [0.16 , 6.80]
Lee A et al. 2017	18	61	18	60	68.2%	0.98 [0.57 , 1.70]
Madaria E et al. 2018	3	19	9	21	22.5%	0.37 [0.12 , 1.16]
**Total (HKSJ[a])**		**103**		**105**	**100.0%**	**0.79 [0.23 , 2.79]**
Total events:	23		29			

Test for overall effect: T = 0.79, df = 2 (P = 0.51)
Test for subgroup differences: Not applicable
Heterogeneity: Tau² (DL[b]) = 0.05; Chi² = 2.34, df = 2 (P = 0.31); I² = 15%

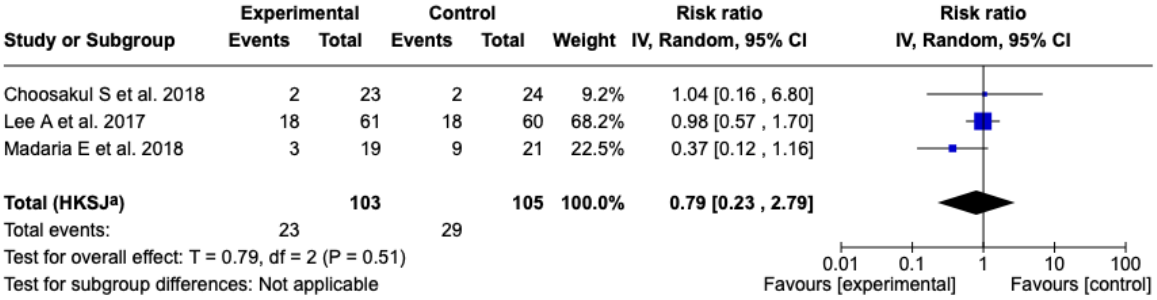

Footnotes
[a]CI calculated by Hartung-Knapp-Sidik-Jonkman method.
[b]Tau² calculated by DerSimonian and Laird method.

Figure S11. Sensitivity analysis of the effect of Ringer's lactate versus normal saline on SIRS at 72 hours in patients with acute pancreatitis, modifying the meta-analysis model.

Study or Subgroup	Experimental Events	Experimental Total	Control Events	Control Total	Weight	Risk ratio IV, Random, 95% CI
Karki B et al. 2022	3	19	7	21	24.5%	0.47 [0.14 , 1.58]
Lee A et al. 2017	11	61	14	60	71.3%	0.77 [0.38 , 1.56]
Madaria E et al. 2018	0	26	3	25	4.2%	0.14 [0.01 , 2.53]
**Total (HKSJ[a])**		**106**		**106**	**100.0%**	**0.64 [0.20 , 2.04]**
Total events:	14		24			

Test for overall effect: T = 1.66, df = 2 (P = 0.24)
Test for subgroup differences: Not applicable
Heterogeneity: Tau² (DL[b]) = 0.00; Chi² = 1.59, df = 2 (P = 0.45); I² = 0%

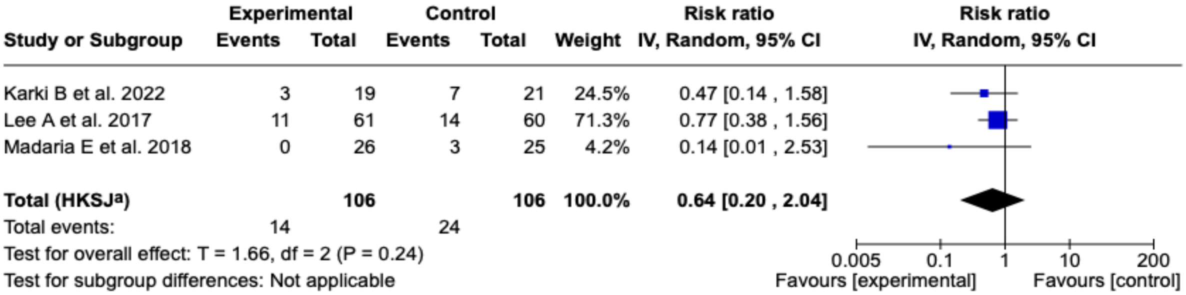

Footnotes
[a]CI calculated by Hartung-Knapp-Sidik-Jonkman method.
[b]Tau² calculated by DerSimonian and Laird method.

Figure S12. Sensitivity analysis of the effect of Ringer's lactate versus normal saline on 48-hour CRP levels in acute pancreatitis, modifying the meta-analytic model.

Study or Subgroup	Experimental Mean	Experimental SD	Experimental Total	Control Mean	Control SD	Control Total	Weight	Std. mean difference IV, Random, 95% CI
Choosakul S et al. 2018	12.1	0.7	23	23.1	4.2	24	58.3%	-3.56 [-4.50 , -2.61]
Madaria E et al. 2018	45.7	20	19	172.7	33	21	41.7%	-4.51 [-5.72 , -3.30]
**Total (HKSJ[a])**			**42**			**45**	**100.0%**	**-3.95 [-9.92 , 2.01]**

Test for overall effect: T = 8.42, df = 1 (P = 0.08)
Test for subgroup differences: Not applicable
Heterogeneity: Tau² (DL[b]) = 0.15; Chi² = 1.48, df = 1 (P = 0.22); I² = 32%

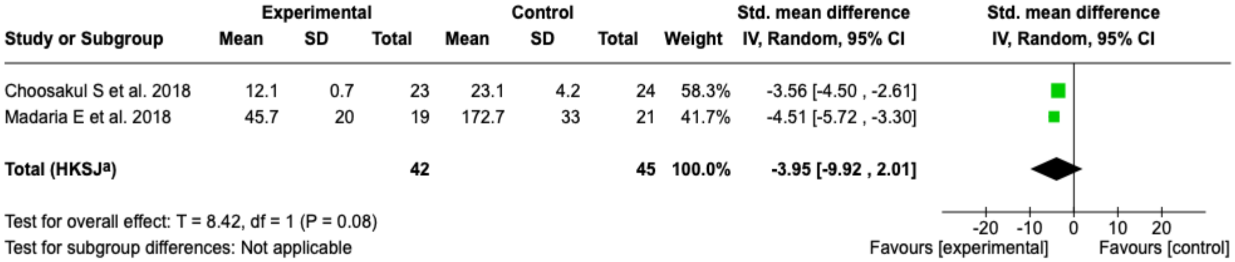

Footnotes
[a]CI calculated by Hartung-Knapp-Sidik-Jonkman method.
[b]Tau² calculated by DerSimonian and Laird method.

Figure S13. Sensitivity analysis of the effect of Ringer's lactate versus normal saline on 72-hour CRP levels in acute pancreatitis, modifying the meta-analytic model.

	Experimental			Control				Std. mean difference
**Study or Subgroup**	**Mean**	**SD**	**Total**	**Mean**	**SD**	**Total**	**Weight**	**IV, Fixed, 95% CI**
Karki B et al. 2022	14.2	0.7	26	23.3	2	25	40.2%	-6.03 [-7.36 , -4.69]
Madaria E et al. 2018	55.5	27.7	19	204	44	21	59.8%	-3.91 [-5.01 , -2.82]
**Total**			**45**			**46**	**100.0%**	**-4.77 [-5.61 , -3.92]**

Test for overall effect: Z = 11.02 (P < 0.00001)
Test for subgroup differences: Not applicable
Heterogeneity: Chi² = 5.75, df = 1 (P = 0.02); I² = 83%

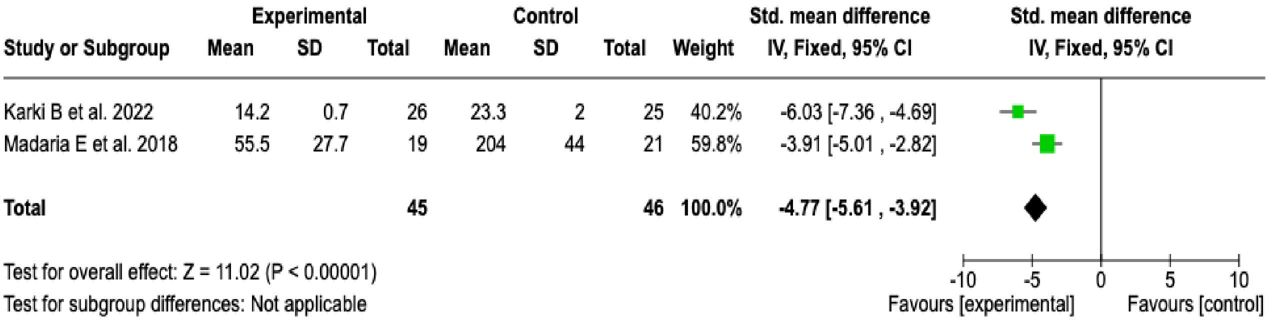

Supplement: Supplementary file 1 [file diseases-13-00300-s001.zip › Figure S1-13.pdf]
